# Antioxidants in brain tumors: current therapeutic significance and future prospects

**DOI:** 10.1186/s12943-022-01668-9

**Published:** 2022-10-28

**Authors:** Xuchen Qi, Saurabh Kumar Jha, Niraj Kumar Jha, Saikat Dewanjee, Abhijit Dey, Rahul Deka, Pingal Pritam, Kritika Ramgopal, Weiting Liu, Kaijian Hou

**Affiliations:** 1 Department of Neurosurgery, Shaoxing People’s Hospital, Shaoxing, 312000 Zhejiang China; 2 Department of Neurosurgery, Sir Run Run Shaw Hospital, Zhejiang University School of Medicine, Hangzhou, 310020 Zhejiang China; 3 Department of Biotechnology, School of Engineering & Technology, Sharda University, Greater Noida, Uttar Pradesh 201310 India; 4 Department of Biotechnology Engineering and Food Technology, Chandigarh University, Mohali, 140413 India; 5 Department of Biotechnology, School of Applied & Life Sciences (SALS), Uttaranchal University, Dehradun, 248007 India; 6 Advanced Pharmacognosy Research Laboratory, Department of Pharmaceutical Technology, Jadavpur University, Kolkata, West Bengal 700032 India; 7 Department of Life Sciences, Presidency University, 86/1 College Street, Kolkata, West Bengal 700032 India; 8 School of Nursing, Anhui University of Chinese Medicine, Hefei, 230001 Anhui China; 9 School of Public Health, Shantou University, Shantou, 515000 Guangdong China

**Keywords:** Antioxidant, Brain tumor, Chemopreventive role, Chemotherapy, Dietary antioxidants, Gene mutation, Glioma, Glioblastoma, Metabolic reprogramming, Oxidative stress

## Abstract

Brain cancer is regarded among the deadliest forms of cancer worldwide. The distinct tumor microenvironment and inherent characteristics of brain tumor cells virtually render them resistant to the majority of conventional and advanced therapies. Oxidative stress (OS) is a key disruptor of normal brain homeostasis and is involved in carcinogenesis of different forms of brain cancers. Thus, antioxidants may inhibit tumorigenesis by preventing OS induced by various oncogenic factors. Antioxidants are hypothesized to inhibit cancer initiation by endorsing DNA repair and suppressing cancer progression by creating an energy crisis for preneoplastic cells, resulting in antiproliferative effects. These effects are referred to as chemopreventive effects mediated by an antioxidant mechanism. In addition, antioxidants minimize chemotherapy-induced nonspecific organ toxicity and prolong survival. Antioxidants also support the prooxidant chemistry that demonstrate chemotherapeutic potential, particularly at high or pharmacological doses and trigger OS by promoting free radical production, which is essential for activating cell death pathways. A growing body of evidence also revealed the roles of exogenous antioxidants as adjuvants and their ability to reverse chemoresistance. In this review, we explain the influences of different exogenous and endogenous antioxidants on brain cancers with reference to their chemopreventive and chemotherapeutic roles. The role of antioxidants on metabolic reprogramming and their influence on downstream signaling events induced by tumor suppressor gene mutations are critically discussed. Finally, the review hypothesized that both pro- and antioxidant roles are involved in the anticancer mechanisms of the antioxidant molecules by killing neoplastic cells and inhibiting tumor recurrence followed by conventional cancer treatments. The requirements of pro- and antioxidant effects of exogenous antioxidants in brain tumor treatment under different conditions are critically discussed along with the reasons behind the conflicting outcomes in different reports. Finally, we also mention the influencing factors that regulate the pharmacology of the exogenous antioxidants in brain cancer treatment. In conclusion, to achieve consistent clinical outcomes with antioxidant treatments in brain cancers, rigorous mechanistic studies are required with respect to the types, forms, and stages of brain tumors. The concomitant treatment regimens also need adequate consideration.

## Introduction

Brain tumors comprise a diverse group of subtypes and they rank among the deadliest forms of cancer. Although they are not uncommon in adults, brain tumors are the most prevalent solid tumors in children and a substantial source of morbidity and death in young people [1]. Despite the fact that brain tumors are rare, they represent very poor survival rates [2]. The unique microenvironmental and intrinsic cell properties of brain tumors have made them practically resistant to majority of conventional and cutting-edged therapies [3]. Numerous strategies for treating brain cancer have been developed but only a few clinically approved drugs exist for medicinal use, thus leaving room for promising treatment modalities. The present-day research also focuses on using antioxidants for the chemoprevention of different types of cancer, including brain cancer, as antioxidants may reduce tumor growth and interfere with carcinogenesis [4].

Antioxidants have been shown to interact with and neutralize free radicals and defuse their effects, which include gene mutation, oxidative damage to chromosomes and proteins, lipid peroxidation of cellular membranes, and dysfunctional cell growth [5]. The brain consists of 20% of the total metabolic activity of the body and has a higher consumption of oxygen than other tissues [6, 7]. Thus, it is thought to be a possible site for oxidative stress (OS) damage, which may encourage the development of brain cancer. Genomic instability caused by OS-mediated damage to cellular macromolecules encourages the development of cancer. Reactive oxygen species (ROS)-sensitive cell signaling events are implicated in the proliferation, growth, differentiation, metabolism, inflammation, angiogenesis, and survival of cancer cells. A growing body of evidence have also revealed the role of ROS in promoting drug resistance in brain tumors [8]. The antioxidant capacity of the brain is significantly lower than other tissues [9]. Endogenous antioxidants involve heterogeneous groups that are water or lipid-soluble enzymes such as superoxide dismutase (SOD), catalase (CAT), glutathione peroxidase (GPx), glutathione reductase (GR), glutathione S-transferase (GST), etc., and some cellular metabolites like reduced glutathione (GSH) and thioredoxin [10]. Various studies have reported that antioxidant systems such as SOD and thioredoxin play a key role in protecting cells from ROS-induced damages [11–13].

Currently, we question whether antioxidants are able to prevent cancer. Different studies and clinical trials have shown different results, which creates a dilemma for researchers. A study showed that the addition of antioxidants, vitamin E, and N-acetylcysteine (NAC) substantially escalated lung tumor formation in mice. Another study claimed that antioxidants treatment promotes lung cancer by endorsing cell proliferation via decreasing ROS level, DNA damage and inactivation of p53 gene expression [14]. A similar report also concludes an increase in the formation of lymph node metastases in mice when supplemented with NAC in drinking water [15]. Some studies have also shown that patients diagnosed with glioblastoma multiforme (GBM) had a higher rate of mortality and a high concentration of vitamin E isoforms (α-tocopherol and γ-tocopherol) in their serum samples [16, 17]. On the other hand, the chemopreventive roles of antioxidants have also been reported elsewhere [4, 8, 11–13]. Thus, the precise role of antioxidants in chemoprevention is still unclear. Exogenous antioxidants also support prooxidant chemistry that is associated with the killing of cancer cells by induction of ROS in excess. Thus, antioxidants could not only be useful in preventing cancer recurrence but also may supplement cancer chemotherapy. The therapeutic dose determines whether effects rendered will be pro- or antioxidant. It is thought that exogenous antioxidants may be helpful for chemotherapeutic purposes at pharmacological/high doses, however dietary amounts may be more useful as chemoprevention. Thus, an antioxidant can be exploited for both tumor chemotherapy and prevention of tumor recurrence depending on the clinical purpose and objective. For this review, we analyzed the knowledge associated with antioxidants and their relationship with brain tumors. We also discussed the therapeutic roles of different antioxidants along with their protective mechanisms for both chemopreventive and chemotherapeutic purposes. Finally, we tried to hypothesize the association between governing factors that regulate the anticancer effect of antioxidants based on the clinical objectives in the management of brain cancer.

## A glance at the United States Food and Drug Administration-approved agents for brain tumor management

Despite the fact that numerous strategies for treating brain cancer have been investigated, only a few drugs have been approved by the United states Food and Drug Administration (FDA) so far for clinical uses (Table 1), thus leaving room for promising treatment modalities [18, 19]. Temozolomide (TMZ) is the most often used and a successful alkylating chemotherapeutic drug for gliomas. A study revealed that TMZ can induce mutational load to a subset of gliomas that endorse tumor recurrence [20]. Lomustine, chemically known as chloroethyl-cyclohexyl-nitrosourea (CCNU), is a monofunctional alkylating agent that is also a standard drug for GBM. It is one of the most effective drug in the polychemotherapy regimen. However, its effectiveness is limited in the treatment of oligodendrogliomas or O^6^-methylguanine-DNA-methyltransferase (MGMT) promoter-methylated GBM. A proper dose is often restricted by toxicities including thrombocytopenia and hematotoxicity [21]. Another alkylating drug, carmustine, chemically known as 1,3-bis(2-chloroethyl)-1-nitrosourea (BCNU), is used for both glioma diagnosis and prevention of tumor recurrence. It is administered either intravenously or by surgical implantation of a BCNU wafer. BCNU treatment has been shown to be effective in seminal trials, but its safety is still debatable. BCNU wafer showed effectiveness in prolonging the overall survival of GBM patients but only for 2–4 months. Its major toxicities include pulmonary fibrosis, hematotoxicity, emesis, etc. [22]. Bevacizumab (BVZ) is a humanized therapeutic antibody that can bind to and suppress vascular endothelial growth factor (VEGF) to inhibit vascular permeability and angiogenesis in tumor cells. It has shown effectiveness in delaying tumor progression in recurrent brain tumor patients but has limited benefits on overall survival in real-world clinical setting [23]. BVZ in combination with other cytotoxic drugs like carboplatin and etoposide also showed therapeutic benefit in clinical trials but is yet to be approved by FDA. While BVZ is well tolerated, suppression of VEGF activity often results in some common toxicities, such as hypertension, gastrointestinal perforation, thromboembolic events, cerebral haemorrhage, problems with wound healing, and proteinuria etc. [19]. In addition to the aforementioned drugs, the FDA has also approved a portable device called Optune that disrupts cancer cell division by generating an electric fields called tumor treating fields (TTFields). Maintenance of GBM patients with wearable Optune device after radiation therapy and chemotherapy (with TMZ) not only increases overall life-span but also improves quality of life. Since TTFields are applied locally to the head, the systemic toxicity is limited [24]. However, in addition to various physiological changes in the brain and burn in the tissues beneath the device placement area, the uncertainty of thermal and electrical behaviors of brain tissues due to the temperature supply by TTFields require serious monitoring when calibrating the device [25].

**Table 1:** FDA-approved agents for the treatment of brain tumor (Adapted from current treatments for brain tumors, National Brain Tumor Society, The United States) [18, 19]

Agents	Manufacturers and year of approval	Drug types	Drug uses	Mechanisms	Side effects
Temozolomide (TMZ)	Celon Laboratories Ltd. 2005	Nonspecific alkylating agent	All high-grade gliomas (HGG) (SOC)	Causes mismatch repair in DNA via methylation of guanine at the O^6^ position	Thrombocytopenia (12%), leukopenia (7%), neutropenia (7%), hematologic toxicity (16%),
Lomustine (CCNU)	Bristol-Myers Squibb Co. 1976	Nonspecific alkylating agent	Recurrent HGG	Facilitates crosslinking of DNA and RNA in dividing cells triggering cell death	Hematologic toxicity (49.7%)
Carmustine (BCNU)	Bristol-Myers Squibb Co. 1977	Nonspecific alkylating agent	Recurrent HGG	Facilitates crosslinking of DNA and RNA in dividing cells; binds to and modifies GR	Ocular toxicity (> 10%), pulmonary toxicity (< 30%), and bone marrow suppression (> 10%)
BCNU wafer implants	Eisai Inc. 1996 & 2003	Nonspecific alkylating agent	Recurrent and new HGG	Causes the crosslinking of DNA and RNA in dividing cells; binds to and modifies GR	Intracrania infection (1–10%), cerebral edema (1–10%), wound-healing complications (12%),
Bevacizumab (BVZ)	Genentech, Inc. 2009	Targeted therapeutic antibody	Recurrent HGG	Binds to and inhibits the VEGF protein in tumor cells	Thromboembolic events (3.2–11.9%), hypertension (5.5–11.4%), gastrointestinal perforation (1.5–5.4%), wound-healing complications (0.8–3.3%), cerebral bleeding (2–5.3%), and proteinuria (2.7–11.4%)
Optune device (TTFields)	Novocure. 2011 & 2015.	Low-intensity (1–3 V/cm), intermediate-frequency (200 kHz) alternating electric fields	Recurrent and new HGG	Disrupts tumor cell mitosis	Seizures (7%) and skin toxicity (43%)

## Oxidative stress and brain tumor crosstalk

**Fig. 1:**
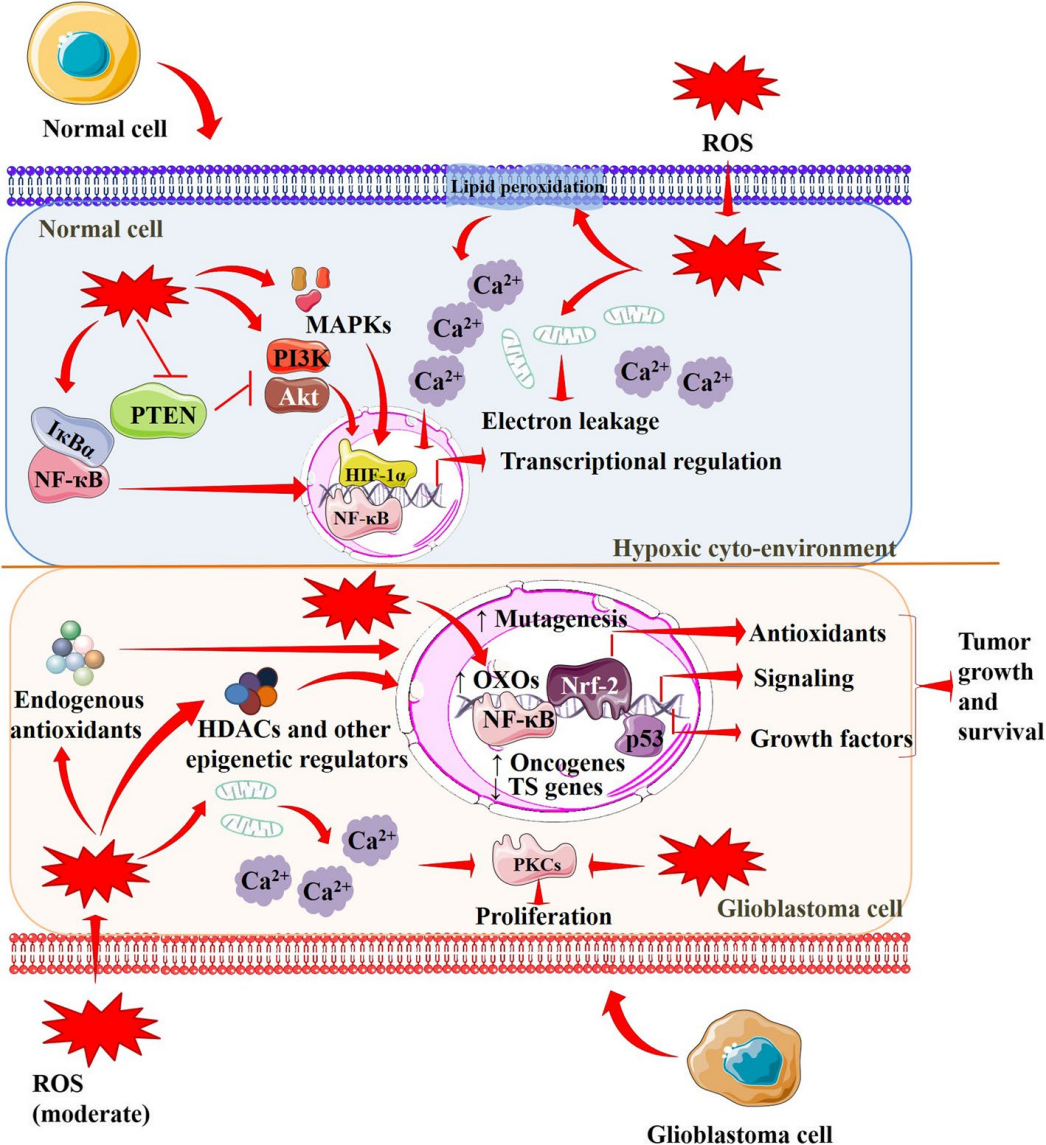
Oxidative stress and redox pathway in normal (above) and cancer cells (below). ROS regulate several signaling events by regulating the transcriptions of different genes in a hypoxic microenvironment that regulate the cell cycle, proliferation, and apoptosis. ROS endorse lipid peroxidation, promote electron leakage, and trigger Ca^2+^ release from intracellular stores. ROS in moderate concentrations activate oncogenes and suppress tumor suppressor genes that in turn increase ROS concentration. Intracellular free Ca^2+^ triggers PKC activation that promotes proliferation. OS also endorses antioxidant genes, promotes angiogenesis, and triggers DNA mutation. In addition, ROS induce HDACs that activate oncogenes and inhibit tumor suppressor genes through epigenetic regulation. Red arrows indicate downstream events and red lines indicate inhibition. Akt, protein kinase B; HDACs, histone deacetylases; HIF-1α, hypoxia-inducible factor 1-alpha; IҡBα, nuclear factor of kappa light polypeptide gene enhancer in B-cells inhibitor, alpha; MAPKs, mitogen-activated protein kinases; NF-κB, nuclear factor kappa-light-chain-enhancer of activated B cells; Nrf-2, nuclear factor erythroid 2-related factor 2; OXO, oxalate oxidase; PI3K, phosphoinositide 3-kinases; PKC, protein kinase C; PTEN, phosphatase and tensin homolog; ROS, reactive oxygen species; TS genes, tumor suppressor genes

The role of OS in oncogenesis at different phases of tumor development and progression has been investigated [26]. OS develops due to a disproportion between the synthesis and accumulation of the free radicals referred to as ROS and reactive nitrogen species (RNS). The ROS species O^2•−^ (superoxide anion), OH^•^ (hydroxyl radical), and H_2_O_2_ (hydrogen peroxide) are formed by the partial reduction of oxygen and the cellular ROS are formed by endogenous mechanisms like the one in mitochondrial oxidative phosphorylation [27]. Nitric oxide (NO), which is also produced by the mitochondria, interacts with O^2•−^ to produce ONOO^•−^ (peroxynitrite). This and other nitrogen-containing free radicals are classified as RNS [27, 28]. Small amounts of ROS are necessary for body cell homeostasis and redox signaling (Fig. 1). At the physiological concentration, ROS regulate signal transduction, gene expression, enzyme activation, and protein folding [29]. Once the threshold levels of ROS are reached, OS conditions arises in the body. A wide range of activities, including oncogene activation, metabolism enhancement, and mitochondrial dysfunction, are connected to the increased intrinsic free radical concentrations [26]. In addition, OS brings about free radical-induced alterations in the DNA, leading to genomic instability. OH^•^ can react/bind with purine and pyrimidine bases of nucleic acids and chromatin proteins, resulting in genomic instability and chromosome modifications that alter the expressions of different genes. Free radical accumulation also decreases endogenous antioxidants. The genomic changes in tissues coupled with decreased levels of cellular antioxidants demonstrate the potential carcinogenic and mutagenic effects. Cancer cells constantly control the response to OS and the generation of ROS, especially H_2_O_2_, for survival and invasion [30].

Several studies have revealed the association between OS and brain tumor development. The brain comprises 2% of the entire body, yet it consumes 20% of total body’s oxygen, representing the possibility of more free radical production compared to other organs [31]. When the brain does not get the oxygen it requires, cerebral hypoxia ensues. Areas of hypoxia increase ROS concentrations in the brain and promote tumorigenesis. Hypoxia activates adaptive cellular activities in both pathological and physiological conditions by inducing endoplasmic reticulum (ER) stress and hypoxia-inducible factors (HIFs) activation [32]. ROS are known to stabilize HIFs in the hypoxic milieu in the brain [33]. ROS-mediated HIF activation is implicated in tumorigenesis in the brain. HIFs can also trigger ROS formation via reduced nicotinamide adenine dinucleotide phosphate (NADPH) oxidase activation. Activation of HIF-1α and HIF-1α (EPAS1) expressions in glioblastoma stem-like cells has been observed [32]. HIFs can simultaneously trigger ROS formation via NADPH oxidase activation. HIFs can regulate several factors including VEGF, pyruvate dehydrogenase kinase 1 (PDK1), insulin-like growth factor 2 (IGF-2), carbonic anhydrase 9, glucose transporter 1, 3 (GLUT1/3), which are involved in cell proliferation, survival, angiogenesis, pH regulation, and metabolism. In addition, HIFs have been shown to regulate apoptotic and cell cycle pathways by regulating several transcription factors [33]. In a hypoxic milleu, ROS regulate the transcriptions of different genes that regulate the cell cycle, proliferation, and apoptosis. ROS endorse lipid peroxidation, promote electron leakage, and disrupt calcium homeostasis. Intracellular free calcium ions endorse activation of protein kinases that promote proliferation. ROS endorse transcription factor like nuclear factor erythroid 2-related factor 2 (Nrf-2) that promote survival of cancer cells under high levels of ROS by endorsing secondary antioxidant enzymes and this chemical modification additionally increases the capacity of drug resistance [26]. ROS also cause nuclear factor kappa-light-chain-enhancer of activated B cells (NF-κB) activation which is involved in cell proliferation, invasion and metastasis [34]. In addition, ROS induce histone deacetylases (HDACs) that activate oncogenes and suppress tumor suppressor genes through epigenetic regulation. Thus, the greater the OS-mediated damage, the worse the risk of developing brain cancers becomes (Fig. 1). Studies have also shown that brain glioma patients exhibit enhanced oxidative damage [5, 26, 33]. Thus, antioxidants could serve as potential chemopreventive agents against brain cancer.

## Oxidative stress biomarkers in patients with brain tumors

**Fig. 2:**
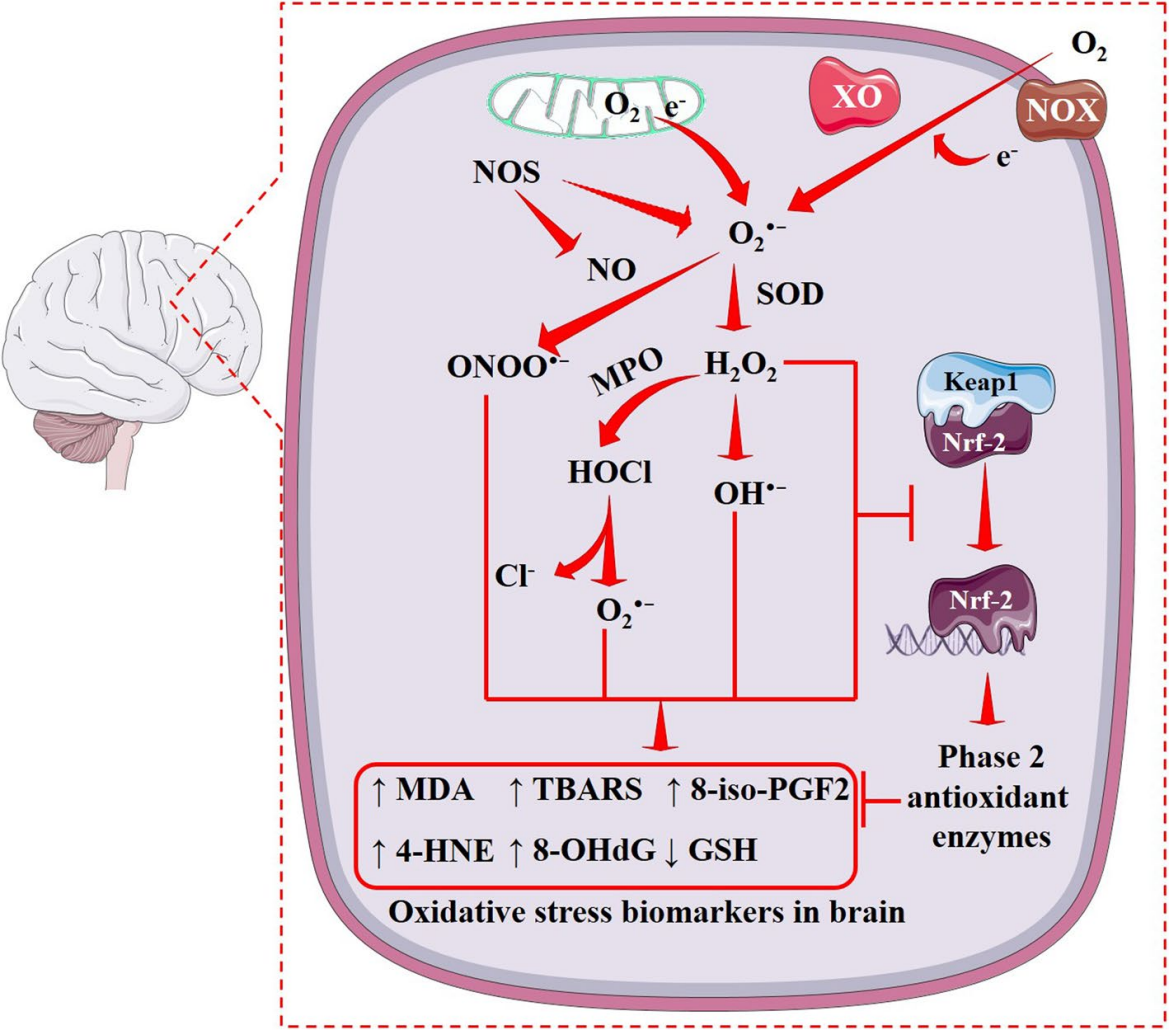
Oxidative stress-related biomarkers in the brain. The mitochondrion is considered to be the major intracellular production house of ROS. Several enzymes, such as NOX, XO, and uncoupled form of NOS can trigger O_2_^•−^ production. NOS also catalyze the metabolic reaction of amino acid to produce NO. NO reacts with O_2_^•−^ and produces ONOO^•−^, a RNS. O_2_^•−^ is converted into H_2_O_2_ by the action of SOD, which subsequently produces OH^•−^. H_2_O_2_ also yields HOCl by the action of MPO, which again yields O_2_^•−^ and highly active Cl^−^. These highly active free radicals deplete GSH and endorse oxidative damages to cellular lipids, proteins, and DNA, resulting in aberrant levels of some specific biochemical markers in the blood circulation. Thus, these specific biochemical markers could serve as potential OS-related biomarkers. Red arrows indicate downstream events and red lines indicate inhibition. 4-HNE, 4-hydroxynonenal; 8-iso-PGF2α, 8-Epi-prostaglandin F2alpha; 8-OHdG, 8-hydroxydeoxyguanosine; DNA, deoxyribonucleic acid; H_2_O_2_, hydrogen peroxide; HOCl, hypochlorous acid; Keap1, Kelch-like ECH-associated protein 1; MDA, malondialdehyde; MPO, myeloperoxidase; NO, nitric oxide; NOS, nitric oxide synthase; NOX, NADPH oxidase; Nrf-2, nuclear factor erythroid 2-related factor 2; O_2_^•−^, superoxide; OH^•−^, hydroxyl radical; ONOO^•−^, peroxynitrite; ROS, reactive oxygen species; SOD, superoxide dismutase; TBARS, thiobarbituric acid reactive substances; XO, xanthine oxidase

Earlier we discussed the oncogenic role of OS in the brain. A growing body of evidence has shown the association between different OS biomarkars in gliomas. Free radical-mediated damages of lipids, proteins, and nucleic acids are seen during enhanced OS. Thus, the products of lipid peroxidation, protein carbonylation and DNA oxidation can serve as potential biomarkers. Unsaturated fatty acid moieties of membrane lipids are attacked by free radicals, which leads to a self-replicating chain reaction of non-enzymatic lipid peroxidation that produces malondialdehyde (MDA), 4-hydroxynonenal (4-HNE), isoprostains and other compounds. These compounds can serve as the potential biomarkers of lipid peroxidation [5, 35]. MDA is mostly used as an indicator of lipid peroxidation. Thiobarbituric acid reactive substances (TBARS), which are quantified in terms of MDA equivalents, are used to measure the extent of lipid peroxidation. Patients with malignant glioma and meningioma exhibit high MDA levels in their sera and tumor tissues [36, 37]. The peritumoral tissue also shows elevated levels of TBARS in patients with astrocytoma and other high-grade intracranial tumors [38]. 4-HNE-protein adducts were found in astrocytic and ependymal tumors and the degree of lipid peroxidation has been regarded to be proportional to that of the extent of malignancy and neovascularization [39]. ROS can cause oxidative damage of proteins via carbonylation. The extent of protein carbonylation can serve as a redox marker [5]. Enhanced levels of protein carbonyls and advanced oxidation protein products were observed in the sera of primary brain tumor patients [40]. On the contrary, Kumar and colleagues found low levels of protein carbonyl and thiols in the plasma of brain tumor patients compared with healthy subjects [41]. DNA oxidation has been thought to be associated with cancer initiation. The guanine base of DNA is more easily oxidized, and 8-hydroxy-2′-deoxyguanosine (8-OHdG) is the oxidized form of guanine, which serves as potential marker of DNA oxidation. A higher expression of 8-OHdG has been found to be a prognostic indicator in most of the solid tumors [42]. The high-grade glioma patients also show substantially high level of 8-OHdG accumulation in tumor tissue [43, 44]. Human MutT homolog protein 1 (hMTH1), which catalyzes the hydrolysis of oxidized form of purine-nucleoside triphosphates can also serve as a biomarker of oxidative DNA damage. Tumor tissues from high-grade glioma patients exhibited a noticeably high expressions of hMTH1 [44]. Thus, OS markers like MDA, TBARS, 4-HNE, protein carbonyls, 8-OHdG, and hMTH1 may serve as potential biomarkers for brain tumors. In addition to these, endogenous enzymatic antioxidants, such as SOD, CAT, GPx, GR, GST etc. and non-enzymatic antioxidants like GSH may act as potential OS biomarkers for brain tumors. Decreased GSH level is indicative of an increased susceptibility to OS and tumorigenesis. In contrast, some reports claimed that increased GSH level can protect cancer cells by redox defense mechanism and promotes tumor progression [5]. This discrepancy may be due to different stages or forms of brain tumors. In an earlier report, Kudo and colleagues showed that GBM, glioma, germinoma, multiple myeloma, and small-cell carcinoma show low GSH levels, while meningiomas show very high GSH level [45]. Although a few reports show increase in GSH levels in the sera of brain tumor patients [31], majority of studies concluded that brain tumors exhibit low GSH level [38, 46]. Thus, GSH can serve as a biomarker for brain tumors. Regarding levels of endogenous antioxidant enzymes, the observations are highly inconsistent. In addition, these enzymes have different activities depending on the brain tumor types [31, 36, 38, 47–49]. Thus, it is difficult to include them as biomarkers for brain tumors. More studies are required to understand the activities of individual enzymes in the tumor milieu in different forms of brain tumors. These biomarkers have been illustrated in Fig. 2.

## Antioxidants in brain tumors

**Fig. 3:**
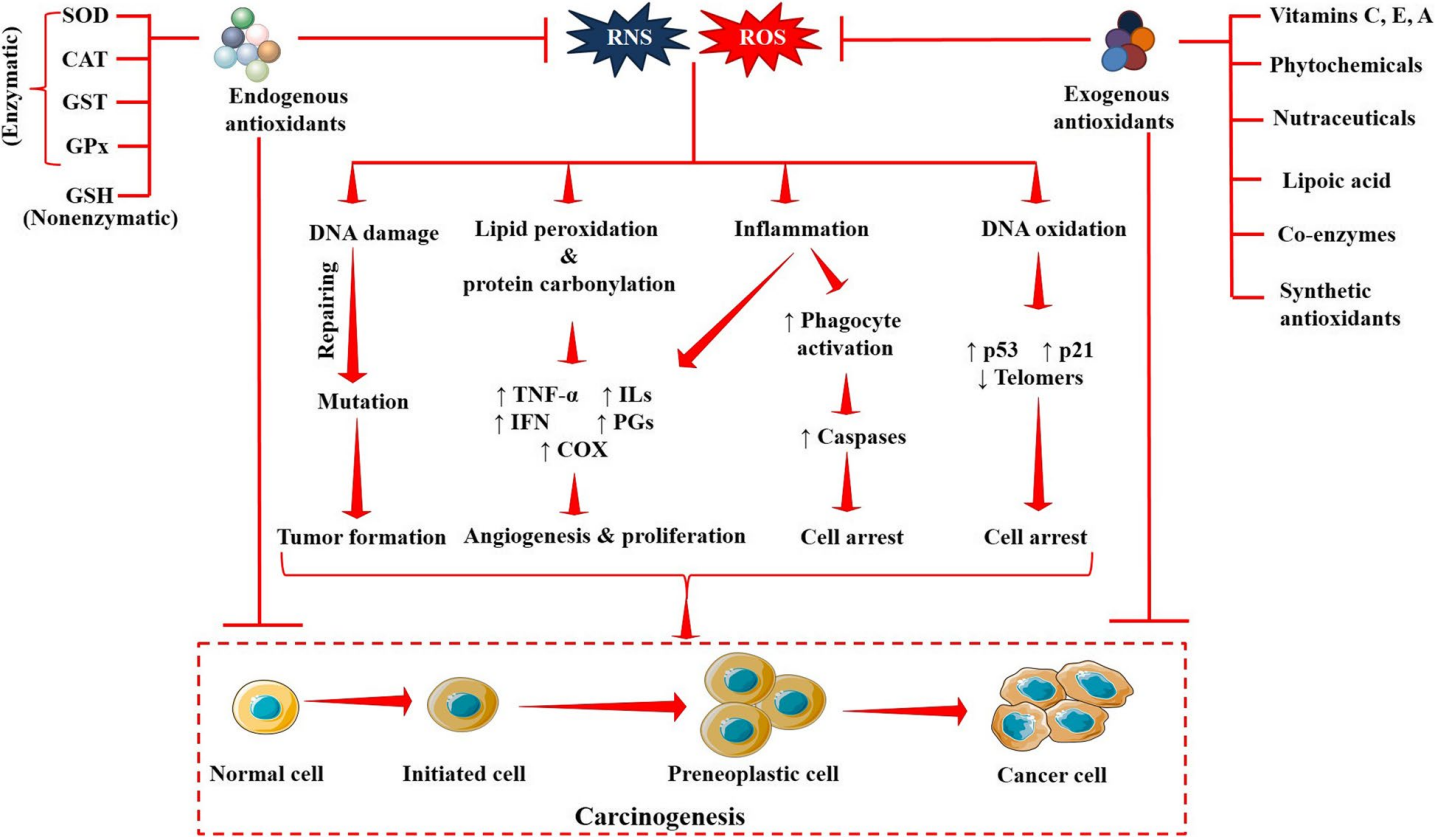
Involvement of oxidative radicals in carcinogenesis and chemopreventive role of antioxidants. ROS and RNS endorse carcinogenesis via DNA damage, DNA oxidation, inflammation, and peroxidative damage of cellular macromolecules, which integrally promote tumor initiation, cell arrest, angiogenesis, and proliferation. Red arrows indicate downstream events and red lines indicate inhibition. CAT, catalase; COX, cyclooxygenase; DNA, deoxyribonucleic acid; GPx, glutathione peroxidase; GSH, glutathione; GST, glutathione S-transferase; IFN, interferon; ILs, interleukines; p21, cyclin-dependent kinase inhibitor p21; p53, cellular tumor antigen p53; PGs, prostaglandins; RNS, reactive nitrogen species; ROS, reactive oxygen species; SOD, superoxide dismutase; TNF-α, tumor necrosis factor alpha

Oxidation is a normal phenomenon of cellular energy metabolism that maintains the functioning of cells. Aerobic metabolism results in the production of ROS, which can then endorse the production of other free radicals like RNS [29]. In normal physiological conditions, the intracellular free radical levels are maintained with the involvement of endogenous antioxidants and other defense components. Endogenous antioxidants comprise heterogeneous groups of compounds including enzymes such as SOD, CAT, GPx, GR, GST, etc. and some cellular compounds like GSH and thioredoxin [10]. Three isoforms of SOD have been recognized so far, which are cytosolic copper and zinc-containing SOD (Cu-Zn SOD), manganese-requiring mitochondrial SOD (Mn SOD) and an extracellular Cu-Zn-containing SOD (EC-SOD). SOD catalyzes the dismutation of O^2•−^ into H_2_O_2_ and O_2_. CAT prevents the cells from the destructive effect of H_2_O_2_ by catalyzing its conversion into H_2_O and O_2_. Both, GST and GPx endorse the radical-scavenging reaction of GSH; while GR rescues GSH by endorsing the conversion of GSSG (glutathione disulfide) into GSH, which forms during radical scavenging reactions of GSH [50, 51]. However, the equilibrium between the ROS and oxidation status deviates in various pathological conditions, such as cancer. Generation of excessive free radicals coupled with depletion of cellular redox defense system disrupts the normal redox cycle, resulting in enhanced OS. The brain tissue requires more oxygen and higher energy than other organs to support normal physiological processes. Additionally, the brain tissue represents high levels of fatty acids and low levels of endogenous antioxidants. These factors collectively make the brain more susceptible to OS [52]. Emerging evidence shows that free radicals regulate many cellular events that endorse oncogenesis by boosting initiation, proliferation, angiogenesis, invasion, and cell arrest, as illustrated in Fig. 3 [26]. Antioxidants are the molecules that protect human cells against free radicals. A regular intake of antioxidant vitamins, such as vitamin C and E during pregnancy reduces the risk of developing pediatric brain tumors [53, 54]. Total endogenous antioxidant levels and malignancy grades in brain tumors have also been found to be inversely correlated. Studies showed that antioxidants could suppress or even arrest the onset of different cancers including brain tumors [5, 54, 55]. Almost all antioxidants have the potential to act as prooxidants in certain concentrations, which trigger different cell death pathways mediated through enhanced ROS production [56, 57]. Thus, the chemotherapeutic effect of antioxidants simultaneously exists. Patients with malignant gliomas (grade III) exhibited higher survival rates when they had significant vitamin E intake [54]. In addition, different antioxidants simultaneously exhibit multiple therapeutic attributes that aid in brain tumor management by promoting radiosensitization, potentiating the effect of chemotherapeutic agents as adjuvants, and reversing chemoresistance [58–60]. Antioxidant mechanism is principally involved in attenuating carcinogenesis; however, they could also be advantageous in cancer management due to their potential chemotherapeutic properties (prooxidant mechanism), function as adjuvant, promotion of radiosensitization, or ability to reverse chemoresistance. Different types (exogenous and endogenous) of antioxidants with their potential healthpromoting attributes with special emphasis on tumor inhibitory effect are presented in Table 2.

**Table 2:** List of antioxidants with potential therapeutic effects against brain tumors

Antioxidants	Chemical structures	Functions
Vitamin A		It is essential for human body functioning; it protects against DNA damage and OS; it acts as an antioxidant and exhibits capacity to relieve oxidation stress; and it prevents the risk of tumor formation.
Vitamin C		It is essential for maintaining proper functioning of various tissues and organs including central nervous system (CNS); it helps in maintaining the metabolism of CNS; it exhibits chemopreventive potential against gliomas; and it acts as an antioxidant and attenuates redox insult.
Vitamin E		It plays several functions in the human body; it acts as an antioxidant and attenuates redox insult; it is effective as chemopreventive agent; and it regulates antioxidant enzymes in various brain tumors.
Curcumin		It protects from developing gliomas; it can eliminate a large variety of cancer cells; it regulates different signaling pathways; it decreases the malignant properties of GBM stem cells by ROS induction (prooxidant property); it endorses autophagy; it reduces metastasis and invasion; it induces G2/M cell cycle arrest phenomenon; and it activates the apoptotic pathways.
Resveratrol		It modulates different signaling pathways; it inhibits viability, proliferation, and migration of cancer cells; it shows ability to accumulate in target organs or cells of tumor location; it acts as a chemosensitizer and increases potential therapeutic efficacy of chemotherapeutic drugs through various mechanisms; it increases ROS level (prooxidant property) in cancer cells; and it induces apoptosis in several cancerous cells via ROS production, increasing mitochondrial membrane permeability, and p53, BAX and caspase activation, etc.
Genistein		It exhibits neuroprotective properties; it exhibits therapeutic properties against brain, bone, and heart defects, as well as postmenopausal cancers; it induces protection against memory impairment by decreasing OS; it enhances cholinergic neurotransmission; it exhibits antioxidants properties and shows chemopreventive potential; it attenuates neuroinflammation and enhances chemopreventive potential against brain tumor development; it increases the expression of neuroprotective genes (CBP, CREB, IGF-1, BDNF and ERK) and inhibit gene involved in pathological events; and it also exhibits chemotherapeutic potential (prooxidant property) simultaneously.
MnSOD		It scavenges superoxide radicals and prevent tumorigenesis; and it modulates the AP-1-mediated cell proliferation pathways and p53-mediated apoptosis.
Cu-ZnSOD		It scavenges superoxide radicals and prevents tumorigenesis; and it exhibits prooxidant effects of increasing ROS production resulting in induction of OS, which leads to apoptosis activation and tissue injury.
Catalase		It is an antioxidant enzyme that converts H_2_O_2_ to water and molecular oxygen; it prevents tumorigenesis and cell proliferation by reducing OS
GSH		It is an antioxidant cellular metabolite and prevent tumorigenesis; it prevents the redox imbalance; it can modulate different signaling pathways; and it also regulates other cellular functions.

The three-dimensional (3D) crystal structures of Mn-SOD (PDB: 1 PM9), Cu,Zn-SOD (PDB: 2JLP), and catalase (PDB: 7VD9) were retrieved from the Protein Data Bank (PDB) database.

*AP-1* Activator protein 1, *Bax* Bcl2-associated X protein, *BDNF* Brain-derived neurotrophic factor, *CBP* CREB-binding protein, *CNS* Central nervous system, *CREB* cAMP-response element binding protein, *DNA* Deoxyribonucleic acid, *ERK* Extracellular signal-regulated kinase, *H*_*2*_*O*_*2*_ Hydrogen peroxide, *IGF-1* Insulin-like growth factor 1, *ROS* Reactive oxygen species.

### Vitamin A

Vitamin A encompasses a group of fat-soluble naturally occurring retinoids that includes retinol, retinoic acid, and retinyl esters. The human body converts β-carotene into vitamin A. Several body functioning, including cell division, growth, DNA methylation synthesis, immunity, prevention of DNA damage, OS, reproduction, eyesight, etc., are maintained by vitamin A [61]. Vitamin A also plays regulatory roles in neuronal development, dendrite growth, and cognitive attributes [62]. The most important attribute of vitamin A is that it acts as an antioxidant, which could attribute a chemopreventive role against tumorigenesis. Blood β-carotene levels and β-carotene consumption has been revealed to negatively influence cancer risks [63]. Lv and colleagues concluded that there was an inverse association between dietary intake of vitamin A and the risk of glioma development via a meta-analysis of pertinent literature up until 2015 [64]. Brain tumor patients had significantly low β-carotene level as compared with healthy subjects. In addition, the extent of malignancy in brain tumors was also inversely related to that of β-carotene levels in the brain [55]. Though these studies have explained the negative association between vitamin A intake and the risk of brain tumor formation, the mechanistic insight is yet to be deciphered. Vitamin A is thought to reciprocate compromised retinoid signaling in an early stage of carcinogenesis [65]. All-trans retinoic acid, a retinoic acid analogue, has been found to inhibit the proliferation of glioma cells by activating p53 and promoting cytoplasmic translocation of β-catenin mediated through axin activation [66, 67]. It also reduces the release of pro-inflammatory mediators, which aids in its chemopreventive attribute [68]. Another retinoic acid analogue, 13-cis-retinoic acid exhibited modest therapeutic efficacy in recurrent GBM patients [69]. In contrast, Giles et al. reported that a higher category dietary intake of vitamin A increases the risk of developing glioma in men [70]. Emerging evidence revealed that vitamin A in pharmacological doses or prolonged consumption could promote tumor progression and increase the risk of cancer mortality via a prooxidant effect [62]. A meta-analysis of relevant studies revealed that intake of fruits and vegetables with a good amount of β-carotene along with other micronutrients could offer a protective effect against gliomas [71]. Thus, it could be said that dietary supplement of vitamin A-enriched food would be beneficial against gliomas; however, hypervitaminosis A could worsen brain tumor outcomes.

### Vitamin C

Vitamin C, also known as L-ascorbic acid or ascorbate, is a micronutrient that is used in numerous metabolic processes and is crucial for maintaining the proper functioning of various tissues including the central nervous system (CNS) [72]. The brain and neuroendocrine systems contain the highest concentrations of vitamin C [73]. Vitamin C is the primary antioxidant molecule that maintains redox balance in the brain. This water-soluble vitamin is not only involved in neuronal differentiation, maturation, and survival, but also acts as a cofactor in different enzymatic reactions involved in catecholamine production, collagen synthesis, and HIF-1α regulation [72]. Vitamin C exhibits both chemopreventive and chemotherapeutic roles via antioxidant and prooxidant mechanisms, respectively.

At dietary concentrations, vitamin C exhibits an antioxidant mechanism and prevent tumorigenesis [74]. Vitamin C prevents DNA damage by reducing OS, thereby preventing carcinogenesis. It also prevents metastasis by strengthening anatomical barriers by endorsing collagen synthesis. It interrupts cell survival by suppressing HIF-1α that is required for the survival of cancer cells in a hypoxic milieu. In addition, it could alter the epigenetic and metabolomic profiles of cancer cells and could eradicate cancer stem cells by activating ten-eleven translocation proteins (TETs) and suppressing pluripotency [74]. There seems to be an inverse association between vitamin C intake and the incidences of different types of cancers including gliomas [75–77]. Decreased level of this antioxidant vitamin is also linked to the severity of brain tumor malignancy [54]. A regular supplement of vitamin C during pregnancy was found to reduce the risk of the fetus in developing pediatric brain tumors [53, 54].

**Fig. 4:**
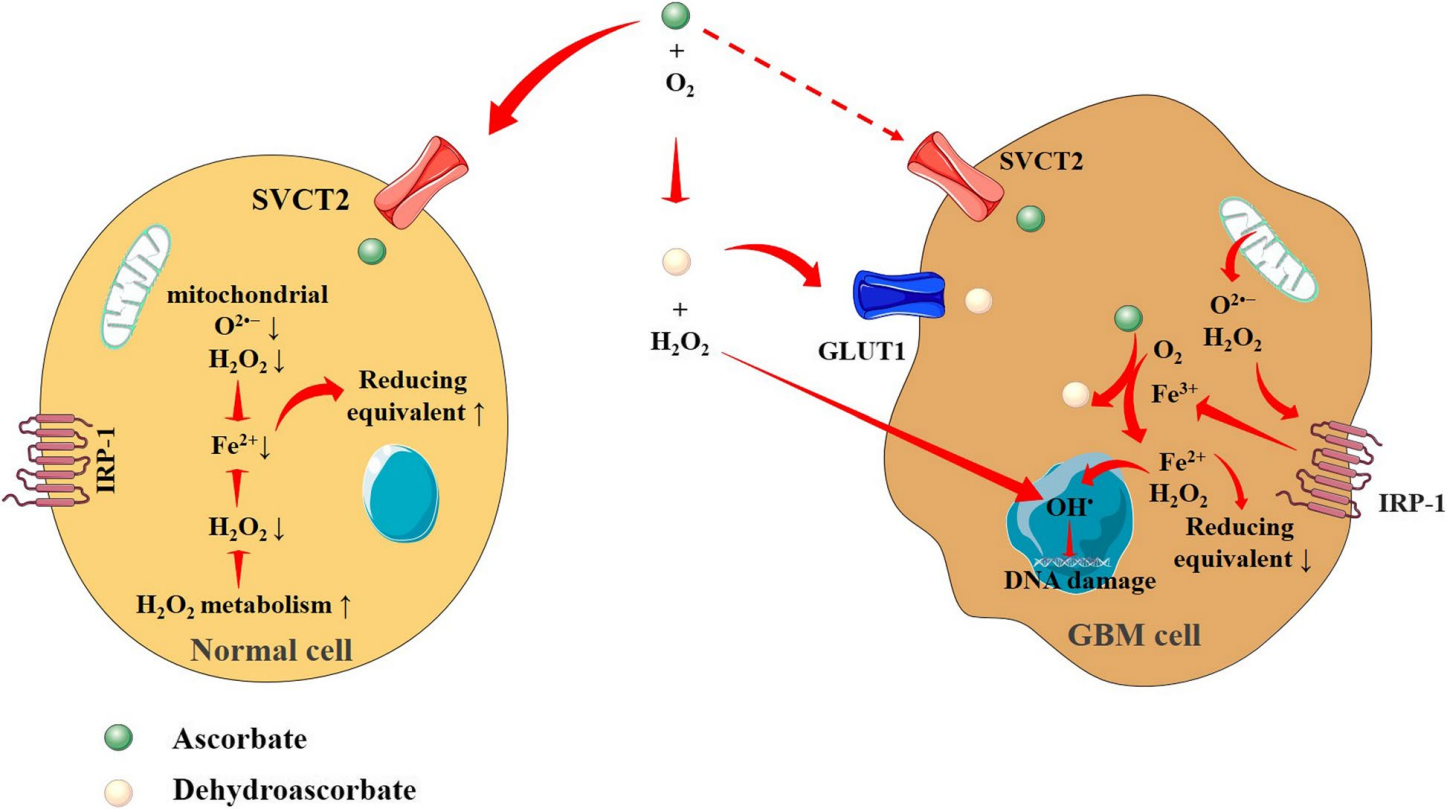
Differential response of ascorbate at a pharmacological dose on GBM cell and normal cell. The difference in H_2_O_2_ metabolism and iron metabolism is the signature of ascorbate selectivity toward cancer cells. Oxidation of ascorbate in the extracellular space produces H_2_O_2_ that enters the cell. In normal cells, ascorbate enters using SVCT2 and exhibits a high ability to metabolize H_2_O_2_, therefore iron metabolism is well maintained resulting in low cellular levels of labile iron (Fe^2+^). In GBM cells, dehydroascorbate enters using GLUT1; however, SVCT2 functionality is changed resulting in a change in the amount of ascorbate within cancer cells (dotted line) which hampers H_2_O_2_ metabolism. Subsequently, there is a build-up of H_2_O_2_ and disturbance in iron metabolism to enhance the levels of labile iron (Fe^2+^) within cancer cells. The intercellular conversion of ascorbate to dehydroascorbate aids in this process. The enhanced accumulation of cellular Fe^2+^ triggers free radical production, as well as decreased redox equivalent resulting in making GBM cells susceptible to vitamin C. Red arrows indicate downstream events and red lines indicate inhibition. ↑ indicates activation and ↓ indicates suppression. GLUT1, glucose transporter 1; H_2_O_2_, hydrogen peroxide; IRP1, iron regulatory protein 1; SVCT2, sodium-dependent vitamin C transporter 2

At pharmacological doses, vitamin C exhibits a prooxidant effect that is required to kill cancer cells [74]. High concentration of vitamin C has shown the potential to kill different cancer cells including GBM cells selectively without affecting normal cells in vitro (Fig. 4) [78, 79]. In preclinical studies, vitamin C monotherapy at millimolar doses exhibits cytotoxic effects on neuroblastoma, glioma, and GBM cells [80–82]. Emerging evidence revealed that vitamin C at pharmacological doses potentiates the formation of O^2•−^ and H_2_O_2_ that disrupt intrinsic iron metabolism and selectively sensitize GBM cells to chemotherapy (Fig. 4) [78]. In vivo assays have shown its potential to reduce the growth of murine glioblastoma tumors by imparting a prooxidant effect [83, 84]. The observations in preclinical studies have been supported by phase I and II clinical trials, where pharmacological doses of parenteral ascorbate demonstrated safety and chemotherapeutic efficacy in prolonging life span and improving quality of life [78, 85]. High-dose vitamin C is becoming popular in palliative care for brain cancer patients due to its efficacy, safety, and tolerability [84]. Various preclinical studies conducted over the past few years have found that high dosages of vitamin C could act as an adjuvant prooxidative agent, primarily in chemotherapy and/or radiotherapy of glioblastoma cells [78, 82, 84, 86]. Vitamin C has been found to enhance the chemotherapeutic effects of methotrexate on glioblastoma cells [87]. According to a case study, a 55-year-old woman with GBM who received both radiotherapy and chemotherapy was given vitamin C infusions (85 g/infusion) 2–3 times/week along with a vitamin C supplement (1 g) before each treatment; this not only improved her quality of life but also halted the progression of her disease [88]. In a first-in-human clinical trial, intravenous ascorbate at pharmacological doses combined with radiotherapy and adjuvant chemotherapy with TMZ assured safety, as well as potential therapeutic efficacy in newly diagnosed glioblastoma patients [89]. A phase II clinical trial is ongoing with 90 GBM patients to study the effectiveness of pharmacological intravenous dose of ascorbate along with radiotherapy and adjuvant chemotherapy with TMZ [90]. However, more studies are required to ascertain the monotherapy- or adjuvant chemotherapeutic efficacy of vitamin C in brain cancer treatment.

### Vitamin E

Vitamin E comes in two different forms that are called tocopherols and tocotrienols and each has four isomers (α, β, γ, and δ). Among them, γ-tocopherol is the most common form of vitamin E in food and α-tocopherol is the predominant form of vitamin E in the blood. This antioxidant vitamin protects highly unsaturated fatty acids in cellular membranes against oxidation. Vitamin E plays a key role in maintaining redox balance in CNS and restores cognitive performance [91**]**. A growing body of evidence supports the tumor inhibitory effect of vitamin E. Vitamin E supplement has been shown to improve the survival of grade III malignant glioma patients [54, 92]. Moreover, a regular intake of vitamin E during pregnancy was found to reduce the risk of developing brain tumors in children [54]. A case-control study comprising 73 glioma and 56 meningioma patients showed that vitamin E exhibited a protective effect [93]. Aggarwal and colleagues found an inverse correlation between serum β-tocopherol and the grade of malignancy in brain tumor [55]. A new prospect of vitamin E is that it could suppress chemotherapy-induced nonspecific organ toxicity and may prolong the life span of cancer patients [94]. Another interesting use of vitamin E and its derivatives is to reverse chemoresistance when included as a component in drug delivery systems [95].

Different epidemiological studies have proposed chemopreventive roles of vitamin E especially γ- and δ-tocopherols, as well as the combination, against different types of cancer. In contrast, α-tocopherol does not appear to have chemopreventive properties and some reports claimed that it often favors carcinogenesis [96, 97]. It has been shown that γ- and δ-tocopherols form short-chain metabolites, γ- and δ-carboxyethyl hydroxychroman and carboxymethylbutyl hydroxychroman, which substantially scavenge ROS and RNS in the cytosol [97]. In addition, γ- and δ-tocopherols more effectively activate peroxisome proliferator-activated receptor gamma (PPARγ) than α-tocopherol and can prevent tumorigenesis [98]. Additionally, γ-tocopherol was shown to induce a cytostatic effect on the cell cycle (G0/G1 arrest) in C-6 glioma cells by suppressing extracellular signal-regulated protein kinase 1/2 and protein kinase C (PKC) upstream and retinoblastoma phosphorylation downstream, leading to the suppression of cyclin E and cyclin-dependent kinase (CDK) 2/4 and activation of p27 [99]. Preclinical studies revealed that γ- and δ-tocopherols inhibit growth, induce apoptosis, and arrest the cell cycle more effectively than α-tocopherol in different cancer cells [100]. Moreover, high α-tocopherol levels could suppress the tumor inhibitory effect of γ- and δ-tocopherols by competing for their binding to the proteins required for chemopreventive effect [97]. γ- and δ-tocotrienols exhibit poor bioavailability but possess better chemopreventive and chemotherapeutic attributes as compared with γ- and δ-tocopherols [101]. In addition, tocotrienols can improve the chemosensitivity of cancer cells toward clinically useful chemotherapeutic agents [102].

In contrast, a metabolomic analysis revealed a positive link between both α- and γ-tocopherols in the sera and the risk of glioblastoma [17]. In a recent report, Yue et al. showed an insignificant positive association between α- and γ-tocopherols in the sera and the risk of glioma [103]. In a survey of 470 glioblastoma patients, Mulpur and colleagues found that complementary therapy with vitamin E non-significantly increases mortality [104]. Recently, Lin and colleagues showed that vitamin E could reverse the effect of the cytotoxic drug bioallethrin on human glioblastoma cells by inhibiting OS and endorsing endogenous redox defense pathways, thus obstructing the chemotherapeutic effects of the drug [105]. However, extensive research and clinical studies are required to reveal the exact regulatory role of individual vitamin E isoforms in brain cancer pathways and to use them in disease management.

### Curcumin

Curcumin, the major bioactive component of turmeric, is a diarylheptanoid, which is a phenolic pigment responsible for the yellow colour of turmeric. It belongs to the group of curcuminoids. Curcumin exhibits several pharmacological attributes that include anti-inflammatory, anticancer, antimutagenic, anti-arthritic, and antioxidant effects [106]. Preclinical studies have shown that curcumin may be effective in the treatment of brain tumors including GBM. On brain tumors, it has been revealed to exhibit both chemopreventive and chemotherapeutic actions; however, it simultaneously protects non-cancer cells through an antioxidant mechanism [107–110]. Curcumin has been shown to induce apoptosis and autophagy, arrest cell cycle (G_2_/M phase), inhibits invasion and metastasis, reduce inflammation, and promote chemosensitization and radiosensitization. In addition, it is clinically safe and non-toxic even at a high therapeutic dose [109]. Curcumin targets several signaling events to attenuate proliferation, survival, and invasion of brain tumor cells and endorse cell death pathways to destroy cancer cells through a pro-oxidant mechanism [108, 111–113]. Curcumin selectively targets human and murine brain tumor cells and induce cell death via p53- and caspase-independent mechanism. It has been proposed that curcumin attenuates cell survival by inhibiting Akt and c-Jun N-terminal kinase (JNK) activation mediated through activator protein 1 (AP-1) and NF-κB suppression [107]. In addition, curcumin exerts tumor suppressive effect via inhibition of E3 ubiquitin ligase NEDD4 (neural precursor cell-expressed developmentally downregulated gene 4) oncoprotein in glioma cells [114]. It can simultaneously arrest the janus kinase (JAK)/signal transducer and activator of transcription 3 (STAT3) pathway to prevent proliferation, invasion, and migration of malignant glioblastoma cells [115]. Emerging evidence revealed that curcumin suppresses STAT3 by reactivating the receptor activator of NF-κB (RANK) via demethylation through epigenetic modification in human glioblastoma cells [116]. In a recent study, the enolase 1 gene (*ENO1*) has been identified as the potential target of curcumin in its antitumor effect against glioblastoma. By suppressing *ENO1*, curcumin inhibits glycolysis, which weakens the energy supply to glioblastoma cells and prevents their proliferation, invasion, and migration [117]. *ENO1* suppression could also be associated with apoptosis induction by curcumin probably mediated through p38 mitogen-activated protein kinase (MAPK) activation and 5′ adenosine monophosphate-activated protein kinase (AMPK) dephosphorylation [117]. Curcumin endorses autophagy by suppressing Akt/mammalian target of rapamycin (mTOR)/p70S6K and activating extracellular-signal-regulated kinase (ERK) signaling in malignant glioma cells. It also protects phosphatase and tensin homolog (*PTEN*) mutation, thereby endorses macroautophagy by supressing Akt/mTOR signaling [118]. Accumulating evidence has revealed that curcumin also prevents tumorigenicity and self-renewal attributes of glioma-initiating cells by inducing autophagy, which prevents the development and recurrence of glioblastoma [119]. Autophagy induction further promotes radiosensitivity of glioma-initiating cells [120]. Despite the fact that curcumin has been proposed to increase the radiosensitivity of CNS cancer cells in various reports, Sminia and colleagues were unable to demonstrate any biological or clinical justification for using curcumin as a radiosensitizer in glioblastoma patients by reviewing all of the literature that has been published up until 2020 [121]. A growing body of evidence showed that micro-RNAs (miRs) could be the mechanistic target of curcumin to exert anticancer and chemosensitizing effects. miR-7, miR-9, miR-21, miR-34a, miR-181, and miR-200c were revealed to be the target miRs of curcumin to impart anticancer effect; while, miR-21, miR-27a, and miR-186 were found to improve chemosensitization toward chemotherapeutic agents [122, 123]. Among them, miR-9 and miR-21 have been shown to induce chemoresistance to different brain cancer cells and thus inhibition of these miRs by curcumin and its analogues could promote the chemotherapeutic effects of anticancer drugs as adjuvants [123–125]. However, bioavailability is an issue with curcumin, which largely challenges the therapeutic efficacy of curcumin against brain tumors. Formulation of curcumin using suitably engineered nanocargo for ensuring targeted delivery could be a solution to overcoming poor pharmacokinetic attributes of curcumin and enhances its therapeutic efficacy in the treatment of brain cancers [126]. So far, we found only one clinical study with a limited number of pre-operative and newly diagnosed glioblastoma patients (*n* = 13). This study demonstrated the prospect of using micellar curcuminoids comprising curcumin (57.4 mg), demethoxycurcumin (11.2 mg), and bis-demethoxycurcumin (1.4 mg) to enhance the bioavailability and intratumoral concentration of curcumin. The study revealed that the intratumoral curcumin concentration may not be sufficient enough to have any immediate anticancer benefits; however, it could aid in long-term tumor growth management. Treatment with micellar curcuminoids 3-times daily for 4 days markedly raised intratumoral inorganic phosphate levels that may be indicative of induction of mitochondrial dysfunction and increased energy metabolism in terms of adenosine triphosphate (ATP) generation [127].

### Resveratrol

**Fig. 5:**
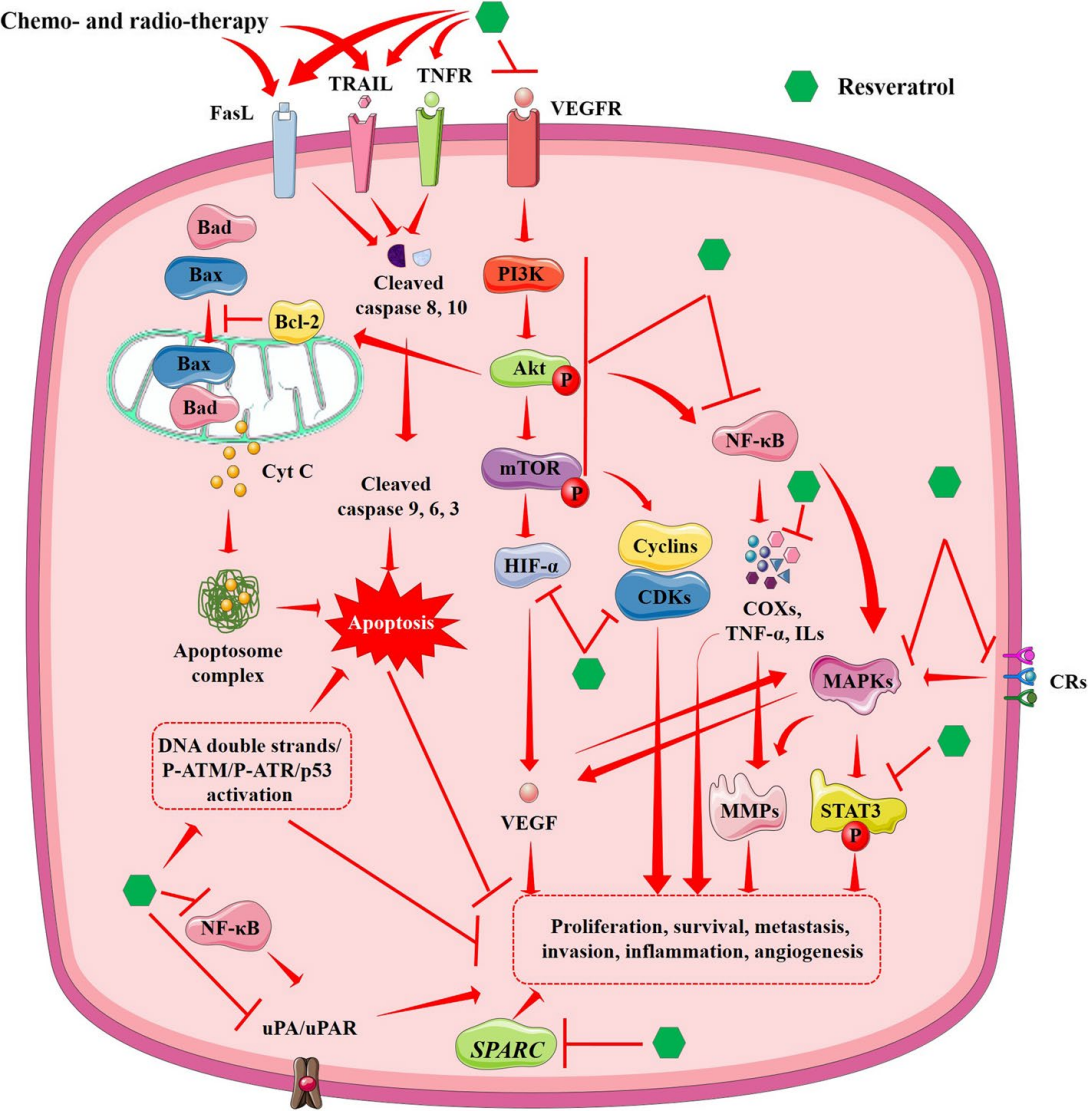
A schematic overview of the mechanism of anticancer effect of resveratrol against brain tumor. Resveratrol exhibits both chemopreventive (via antiproliferative, anti-metastatic, anti-invasive, anti-inflammatory, anti-angiogenic effects) and chemotherapeutic effects (via apoptotic induction) by regulating several signaling events. In addition, it can simultaneously endorse chemo- and radio-sensitization to improve therapeutic efficacy. Red arrows indicate downstream events and red lines indicate inhibition. Akt, Protein kinase B; Bad, Bcl2 associated agonist of cell death; Bax, Bcl2-associated X protein; Bcl-2, B-cell lymphoma 2; CDK, cyclin-dependent kinase; COXs, cyclooxygenases; Cyt C, cytochrome C; ERK, extracellular signal-regulated kinase; FasL, Fas ligand; HIF-α, hypoxia-inducible factor 1-alpha; MAPKs, mitogen-activate protein kinases; MMPs, matrix metalloproteinases; mTOR, mammalian target of rapamycin; NF-κB, nuclear factor kappa-light-chain-enhancer of activated B cells; PI3K, phosphatidylinositide 3-kinases; SPARC, secreted protein and acidic rich cysteine; STAT3, signal transducer and activator of transcription 3; TNF-α, tumor necrosis factor-alpha; TNFR, tumor necrosis factor receptor; TRAIL, Tumor necrosis factor-related apoptosis-inducing ligand; uPA, urokinase plasminogen activator; uPAR, uPA receptor; VEGF, vascular endothelial growth factor; VEGFR, vascular endothelial growth factor receptor

Resveratrol, a stilbenoid polyphenol, is a naturally occurring antioxidant molecule that exhibits a plethora of pharmacological attributes which include antidiabetic, anti-inflammatory, neuroprotective, vasorelaxant, and cardioprotective properties [128, 129]. Resveratrol can successfully cross the blood-brain-barrier (BBB), making it a potential therapeutic or prophylactic agent against CNS-related diseases including brain cancers [60]. Additionally, resveratrol exhibits anticancer properties and prevents carcinogenesis by interfering with initiation, proliferation, invasion, and metastasis [60, 128, 129]. A growing body of evidence revealed that it can prevent carcinogenesis by interfering with initiation, proliferation, invasion, and metastasis. It also inhibits the survival of cancer cells through a proapoptotic mechanism. Emerging evidence revealed the ability of resveratrol to detoxify carcinogens to prevent cancer initiation [130]. Resveratrol simultaneously exhibits chemotherapeutic effects on cancer cells through a prooxidant mechanism that increases ROS production, induces ER stress, encourages apoptosis, and arrests the cell cycle (G_0_/G_1_ and S-G2/M phases) [131, 132]. Resveratrol regulates several signal transduction pathways to exhibit chemopreventive and chemotherapeutic effects in the management of brain cancers (Fig. 5). Resveratrol suppresses STAT3 signaling in glioma cells by inhibiting JAK2 or Src activation, thus resulting in antiproliferative and apoptotic properties [132]. Accumulating evidence shows that resveratrol inhibits some oncogenic miRs, such as miR-19, miR-21, and miR-30a-5p, which is subsequently accompanied by the suppression of their target genes including epidermal growth factor receptor (EGFR), STAT3, cyclooxygenase (COX)-2, and NF-κB, and subduing PI3K/Akt/mTOR signaling [133]. Moreover, it can induce apoptosis and inhibits survival by activating the transcription of tristetraprolin (TTP) in human glioma cells [134]. It blocks NF-κB in the inflammatory tumor microenvironment which subsequently inihibits pro-inflammatory mediators like tumour necrosis factor α (TNF-α) and interleukin (ILs) and enzymes (COX1/2), resulting in the inhibition of tumor progression and metastasis. Resveratrol has been shown to impart chemopreventive effect by down-regulating TNF-α-induced activation of urokinase plasminogen activator (uPA) and uPA receptor (uPAR) mRNA, expressions resulting in the inhibition of human glioma cell invasion [135]. In addition, it demonstrates anti-inflammatory effects by suppressing lymphocyte proliferation. Resveratrol mitigates glioma angiogenesis via inhibition of PKC, matrix metalloproteinases (MMPs) and VEGF. Its anti-metastatic effect is mediated through inhibition of secreted protein and acidic rich cysteine (*SPARC*) gene in glioma cells [132]. Accumulating evidence shows that resveratrol inhibits survival, proliferation, and motility of glioblastoma cells by modulating the Wnt signaling pathway. It was also found to interfere with chemoresistance and dissemination of glioblastoma cells by preventing epithelial-mesenchymal transition (EMT) through Twist1 and Snail1 suppression [136]. Interestingly, resveratrol increases ROS level within cancer cells through a prooxidant mechanism. By interacting with mitochondria, it induces an imbalance in the activities of endogenous antioxidants resulting in an increased accumulation of ROS and lipid peroxides in cancer cells. Increased accumulation of ROS and lipid peroxide can induce OS in glioma cells and endorses apoptosis [60, 137, 138]. Thus, resveratrol can serve as a potential chemotherapeutic agent to treat brain tumors [139].

In addition, resveratrol promotes radio- and chemo-sensitizing potential and exhibits a pivotal role as an adjuvant in standard glioma therapy. Resilience to radiotherapy is a primary cause of the poor prognosis of glioma patients. Preclinical data have shown that resveratrol substantially augments radiosensitization of glioma stem cells in both in vitro and in vivo by inducing autophagy, thus preventing tumorigenesis and tumor recurrence [140]. Resveratrol converses multidrug resistance and sensitizes cancer cells to common chemotherapeutic agents. It improves the therapeutic efficacy of TMZ against glioblastoma by reducing autophagy through suppression of ERK activation using an antioxidant mechanism and subsequently augmenting apoptosis observed in both in vitro and in vivo [141]. In another study, a combination of resveratrol and temozolomide showed synergistic antiproliferative effects. In contrast to earlier observation, the effect is predominantly mediated through additive prooxidant effects of the drugs, resulting in amplified ROS production, AMPK activation, mTOR inhibition, and B-cell lymphoma 2 (Bcl-2) suppression [142]. Resveratrol has also been shown to enhance the chemosensitivity of glioblastoma-initiating cells to temozolomide by endorsing DNA double strand/ phospho-ataxia-telangiectasia mutated (ATM)/phospho-ATM- and Rad3-related (ATR)/p53 and suppressing STAT3 pathways [143, 144]. Emerging evidence showed that resveratrol enforced mitotic disaster and senescence in different TMZ-treated glioblastoma cells, which hampered cell division and increased the chemotherapeutic potential of TMZ [145]. Resveratrol can also enhance the prooxidant and apoptotic effects of paclitaxel by activating the transient receptor potential cation channel, subfamily M, member 2 (TRPM2) channel in glioblastoma cells [60, 146].

However, resveratrol possesses poor water solubility, bioavailability, and stability, which largely compromise its therapeutic efficacy [147]. Formulation of resveratrol employing appropriately tailored nanocargos can be a solution to overcoming the poor pharmacokinetic attributes of the molecule and enhancing its therapeutic efficacy in brain tumor management [60, 132, 148–150]. So far, no clinical data is available in support of the chemotherapeutic, chemopreventive, or radio- and chemo-sensitizing effects of resveratrol on human brain cancers. Thus, suitably designed formulations of resveratrol to fit the specific anticancer mechanisms and improved biopharmaceutical and pharmacokinetic properties are still needed to make resveratrol reasonable for clinical use in brain tumor management.

### Genistein

Genistein is a bioactive isoflavone mainly found in soy and fava beans. Different preclinical studies revealed that it inhibits carcinogenesis by preventing cancer initiation and progression including CNS cancers [151–154]. It exhibits both chemopreventive and chemotherapeutic effects by arresting the cell cycle, suppressing proliferation, inhibiting inflammation, and endorsing apoptosis. Genistein exhibits an anticancer effect with a mechanism comparable to that of resveratrol [154]. Genistein is capable of inducing apoptosis of Bcl-2-silenced malignant neuroblastoma cells by both ligand- (Fas ligand/TNF-related apoptosis-inducing ligand) death receptor- (Fas/TNF receptor) mediated extrinsic and mitochondria-dependent intrinsic pathways [155]. Genistein triggers ER stress and activates calpain 1, which sequentially promotes Bcl-2 associated X-protein (Bax) and BH3 interacting domain death agonist (Bid) cleavage and the translocation to active Bax and t-Bid to mitochondria followed by the cytosolic release of cytochrome C and apoptosome formation. Genistein induces ER stress by activating protein glucose-regulated protein 78 (GRP78) expression, which in turn elicits the CCAAT/enhancer-binding proteins (C/EBPs) homologous protein (CHOP) expression resulting in an induction of apoptosis in cancer cells. Calpain 1 endorses permeabilization of lysosomal membranes and triggers the release of cathepsin B and deoxyribonuclease II to induce apoptosis by activating poly (ADP-ribose) polymerase (PARP) cleavage. Genistein also induces apoptosis by impairing aerobic glycolysis through downregulation of HIF-1α, GLUT1 and/or hexokinase 2 (HK2). In addition, it endorses phospho-ATM/ phospho-ATR/p53/p21 signaling events that are implicated in apoptosis induction and the cell cycle arresting processes. Genistein suppresses TNF-α-induced NF-κB activation as well as nuclear factor of kappa light polypeptide gene enhancer in B-cells inhibitor (IκB) kinase and IκB phosphorylation resulting in an inhibition of inflammation in the tumor microenvironment [154]. Genistein inhibits the proliferation and differentiation of neuroblastoma cells by inducing apoptosis and suppressing both mitogen-activated and intrinsic tyrosine kinase activity and N-myc activation. However, it produces only an insignificant effect on MAPK [151]. It inhibits the growth of GBM and medulloblastoma cells exhibiting variable radio-responses and TP53 mutations by arresting the cell cycle (G_2_/M phase). It simultaneously arrests the growth of GBM and medulloblastoma cells by inhibiting telomerase activity [152]. In addition, genistein can suppress MMP-2 and VEGF expression in both high- and low-grade glioma-derived mesenchymal stem cell-like cells implicating its ability to arrest angiogenesis during cancer progression [156]. It suppresses environmental endocrine disruptor- and estradiol-provoked proliferation of neuroblastoma cells by suppressing Akt phosphorylation [157]. It also inhibits X-ray-induced invasion and migration by suppressing the DNA-protein serine-threonine kinase/Rac/Akt signaling pathways in glioblastoma cells [158]. Genistein has been also found to act as an epigenetic modifier that endorses demethylation of chromodomain helicase DNA binding protein 5 (*CHD5*) and enhances the expressions of *CHD5* and *p53* contributing to growth inhibition and microvessel formation in murine neuroblastoma. Genistein-provoked demethylation of *CHD5* promoter was thought to be associated with DNA-methyltransferase 3 beta suppression [159]. In addition, genistein also improves the effect of chemotherapeutic drugs in brain tumor management. Genistein has been shown to synergize the cytotoxic and antiproliferative effect of carmustine on human GBM and rodent glioma cells [160]. The combination of LC3 silencing and genistein treatment significantly inhibits autophagy and triggers apoptosis in malignant neuroblastoma cells and neuroblastoma xenografted mice. Thus, LC3 inhibition along with genistein therapy could be a suitable therapeutic approach in the management of malignant neuroblastoma [161]. However, the poor pharmacokinetic attributes of genistein limit its clinical applications. Structural modification or novel formulation development are potential solutions for further clinical development of genistein [162]. Polymeric nanoformulation co-loaded with genistein and TMZ has shown promising therapeutic efficacy against GBM and exhibits synergy between genistein and TMZ [163]. A near-infrared-responsive indocyanine-genistein nanoformulation has also been shown to be an effective novel formulation in the combinatorial photo- and chemotherapy of glioblastoma [164]. To the best of our knowledge, genistein has not shown any clinical evidence of an anticancer effect on human CNS cancers [165]; however, a thorough investigation followed by clinical studies would make it possible to employ the therapeutic potential of genistein against human brain cancers.

### SOD

SOD is an endogenous antioxidant enzyme that catalyzes O^2•−^ quenching under both physiological and pathological conditions. It acts as a defense molecule to protect different tissues and organs against redox challenges. Activation of Cu-ZnSOD (SOD1) has been regarded to be neuroprotective [166]. It has been shown to protect the brain by suppressing focal ischemia-induced cerebral apoptosis in mice by suppressing ERK1/2 activation [167]. It is critical to maintaining mitochondrial function in the brain during GSH depletion [168]. A higher concentration of SOD1 in plasma and erythrocytes in brain tumor patients indicates higher OS [31]. Malignant CNS tumors have immunoreactivity to MnSOD (SOD2) in both the intra- and extracellular matrix, despite the fact it is not detected in normal brain tissue [169]. The expression of SOD2 increases with the extent of malignancy in neuroepithelial brain tumors [170]. Higher (⁓45-fold) levels of SOD2 are also observed in cerebrospinal fluid samples from patients with malignant tumors [169]. These observations argue against the therapeutic role of SOD in cancer treatment. In contrast, some reports reveal significant suppression of SOD activity in different brain tumors [5, 48, 54]. This hetorogenicity of observation may be associated with tumor types and/or malignancy stages. However, a growing body of evidence reveals that SOD-based treatments in combination with other chemopreventive drugs and/or radiation can improve therapeutic efficacy in the management of cancers, including brain cancers [171–173]. In a skin cancer model, treatment with MnTE-2-PyP^5+^, a Mn porphyrin-based SOD mimic, following apoptosis but before proliferation significantly arrest multiplicity by suppressing AP-1 expression, protein carbonylation, and proliferating cellular nuclear antigen level without influencing p53 and DNA fragmentation [174]. MnTnBuOE-2-PyP^5+^ enhances carbenoxolone-mediated TNF-related apoptosis-inducing ligand (TRAIL)-induced apoptosis in GBM cells. The enhanced cytotoxic effect could be achieved via production of cytotoxic H_2_O_2_ by dismutation of O^2•−^ [173]. Emerging evidence shows that Mn porphyrin-based SOD mimics can enhance radiation response to cancer cells, while they protect normal cells from radiation damage. Treatment of Mn porphyrin-based SOD mimics along with chemotherapy/radiotherapy/ascorbate treatment enhances therapeutic efficacy by triggering OS and suppressing NF-κB, HIF-1α, AP-1, VEGF, MAPKs, phosphatase 2A, GST, etc. In contrast, Mn porphyrin-based SOD mimic treatment protects normal cells from radiation-induced toxicity by suppressing OS, NF-κB, TGF-β, collagen, and plasminogen activator inhibitor-1 and activating Nrf-2, CAT, MnSOD, NQO1, etc. [172]. MnTnBuOE-2-PyP^5+^ with four escalating doses did not exhibit any adverse reaction in healthy brain tissues of glioma patients treated with concomitant radiation and TMZ in a phase I clinical trial (NCT02655601); this compound qualifies for phase II trials [175].

### CAT

CAT is another antioxidant enzyme that catalyzes the conversion of H_2_O_2_ to H_2_O and O_2_, which saves cells from the harmful effect of H_2_O_2_. Compared to normal brain tissue, brain tumor tissue exhibits considerably less H_2_O_2_ detoxification by CAT. CAT level has been found to be decreased specifically in the nucleus and mitochondria of brain tumor cells [176]. Compared with the control group, patients with various forms of brain tumors showed a statistically insignificant decrease in CAT levels [177]. On the contrary, other reports showed that CAT levels were significantly increased in brain tumor tissue [36, 47, 48]. In a recent report, according to the Cancer Genome Atlas database, glioma tumor tissue represents high CAT mRNA expression compared with normal tissue. In addition, CAT expression is inversely associated with the survival of glioma patients. In glioma cells, CAT overexpression significantly reduces basal H_2_O_2_ level and thus promotes cell growth, inducing resistance against conventional chemo- and radiotherapy [178]. Emerging evidence reveals that membrane-associated CAT imparts resistance and favors the growth of cancer cells. Enhanced ROS formation and altered expression of antioxidant enzymes potentially favor cancer cell proliferation. In the interim, they make cancer cells especially vulnerable to an oxidant attack [179]. Thus, modulation of redox status in brain tumor cells by regulating CAT expression could serve as a potential therapeutic approach in brain cancer management. Antibody-mediated CAT inhibition in vitro has emerged as a promising approach in experimental cancer therapy [180]. Silencing CAT in murine glioma cells was found to increase intracellular ROS and extracellular H_2_O_2_, resulting in improved radiosensitivity; however, pharmacological inhibition of CAT cannot produce an effect on cell viability unless additional OS is induced via oxidants or radiation [181].

### GSH

GSH, γ-l-glutamyl-l-cysteinyl-glycine, is an antioxidant tripeptide that is mainly found in the cytosol of a cell. This low molecular weight thiol plays a vital role in sustaining the intracellular redox balance. GSH with GSH-regulated enzymes constitutes a redox defense system in the brain and exhibits neuroprotective effects. It has a great role in the modulation of enzyme activity, activation of DNA repair, and regulation of transcription factors and different metabolic processes [182, 183]. GSH also plays a key role in the detoxification and elimination of carcinogen and imparts a chemopreventive role [184]. Significant variability of GSH levels was seen in different types of cancers; however, accumulating evidence shows that the majority of brain tumor cases are associated with depletion of GSH levels in brain tumor tissue compared with healthy brain tissue [38, 46]. However, conflicting reports also noted that brain tumor patients showed a higher level of GSH [31]. In a previous report, Kudo and colleagues demonstrated that only meningiomas exhibit extremely high GSH levels, in contrast to other forms of brain tumors, such as GBM, gliomas, germinoma, multiple myeloma, and small-cell carcinoma [45]. Moreover, GSH levels also serve as a prognostic marker of the malignancy of brain cancer. High grade (III/IV) gliomas and astrocytomas exhibit significantly lower GSH levels compared with low grade (I/II) tumors [38, 185]. High levels of ROS production during tumor progression have been implicated in the reduction of GSH and GSH-associated enzymatic activities. Chemotherapeutic drug like 5-fluorouracil can reduce tumor growth by inducing apoptosis but can neither improve redox status nor GSH level in non-primary brain tumor bearing mice [186]. NAC, a GSH precursor has shown promise of inhibiting proliferation, growth, invasion, and migration of glioblastoma cells, as well as of inducing apoptosis by dowregulating neurogenic locus notch homolog protein 2 (Notch2) signaling pathways. The effect seems to be independent of GSH and ROS levels in glioblastoma cells [187]. This report may indicate that enhancement of GSH levels may not be a therapeutic approach in brain tumor treatment. Moreover, emerging evidence revealed the positive association between GSH and chemoresistance in different types of cancers including brain cancers [188, 189]. The chemoresistance in primary brain tumors may arise due to the interplay between multidrug resistance-associated protein-triggered efflux of the drug-GSH conjugate and GST/GSH-provoked drug detoxification [188]. Thus, GSH inhibition may reverse drug resistance to improve chemotherapeutic efficacy. A strategy like using buthionine sulfoximine to directly deplete GSH has been investigated to improve the chemotherapy efficacy in brain cancers; however, lack of selectivity for tumor cells and nonspecific organ toxicity restricts its clinical application [188, 190–192]. Thus, it would not be wrong to mention that GSH acts as a double-edged sword. On the one hand, it inhibits the initiation of cancer by metabolizing carcinogens. On the other hand, its detoxification action restricts the chemotherapeutic effect of drugs and supports the chemoresistance of cancer cells. Inclusion of GSH as a formulation component could aid the therapeutic efficacy of chemotherapeutic drugs. GSH functionalization of a formulation facilitates crossing the BBB mediated through the GSH transporter and may deliver the drug to the brain. GSH-PEG (polyethylene glycol)-ylated liposomal doxorubicin improves therapeutic efficacy by increasing doxorubicin concentration in the brain without altering BBB integrity [193, 194]. Transferrin-targeted GSH-sensitive hyaluronic acid-poly(lactic-co-glycolic acid) nanomicelle loaded with a heat shock protein 90 (Hsp90) inhibitor, AUY922 to enhance the therapeutic efficacy towards brain cancers. GSH conjugation allows fast release of AUY922 to the tumor site and cellular uptake through transferrin receptor [195].

### Other naturally occurring antioxidants in brain tumor management

Flavonoids are the most interesting class of naturally occurring polyphenolic antioxidants that exhibit significant chemopreventive and chemotherapeutic effects in different types of cancers including brain cancers [196, 197]. Flavonoids can target several molecular pathways involved in cell growth, proliferation, inflammation, invasion, survival, angiogenesis, and metastasis processes of tumorigenesis in the brain. They are equally effective in inducing apoptosis by a prooxidant chemistry to exhibit chemotherapeutic effects on brain cancer cells [197–200]. Polyhydroxylated flavonoids, such as quercetin, rutin, apigenin, kaempferol, 3′,4′-dihydroxyflavone, epigallocatechin gallate, and chrysin exhibit the capacity to inhibit migration and invasion, obstruct metabolism, promote differentiation, and induce apoptosis of human glioblastoma cells. These flavonoids have been found to suppress cell migration by modifying the cell surface, reducing filopodia-like structures, downregulating MMP2, and activating fibronectin (both intra- and extra-cellular) and laminin (intracellular) in human glioblastoma cells. They also induce apoptosis by damaging rough ER and mitochondria [201]. Galangin, a galangal flavonoid has been shown to have interesting in vitro and in vivo anti-GBM properties by simultaneous elicitation of apoptosis, pyroptosis, and autophagy. However, pharmacological inhibition of autophagy has been found to enhance galangin-induced apoptosis and pyroptosis in human GBM cells, which proposes an effective therapeutic approach for GBM by a combination of galangin and an autophagy inhibitor [202]. Apigenin exhibits both anti-carcinogenic and chemotherapeutic effects against various types of human malignancies including glioblastoma. It blocks tumorigenesis via protection from carcinogenic stimuli, and by inhibiting tumor cell proliferation, arresting the cell cycle, and inducing apoptosis [203, 204]. It inhibits EMT via endorsing cytoskeleton shrinkage, upregulating E-cadherin activation, and suppressing N-cadherin, snail, and vimentin. Moreover, it can endorse apoptosis by encouraging ROS production mediated through mitochondrial dysfunction, ER stress, and ER stress-mediated protein activation, including phosphorylation of eukaryotic initiation factor 2 (eIF2α), protein kinase RNA-like ER kinase (PERK), CHOP, activating transcription factor 4 (ATF4), and cleaved-caspase 12, thereby inducing apoptosis. Xanthohumol, a prenylated flavonoid has shown promise in suppressing the growth of malignant brain tumor by reducing glucose metabolism via hexokinase 2 inhibition mediated through c-Myc downregulation in glioblastoma cells. Xanthohumol is thought to destabilize c-Myc and promotes its ubiquitination as a consequence, resulting in the suppression of Akt/ flycogen synthase kinase-3 beta (GSK3β) axis and inhibition of glioblastoma cell proliferation. In an in vivo model, it also exhibited tumor suppression in xenograft mice [205]. Flavonoids including quercetin, chrysin, formononetin, epigallocatechin-3-gallate, hispidulin, rutin, icariin, sylibinin, etc. synergistically increase the chemotherapeutic effects of anti-neoplastic drugs in the management of brain cancers [206]. However, bioavailability, BBB permeability, stability, and safety are some of the key issues with flavonoids that largely interfere with therapeutic potential of flavonoids in brain cancer management. Formulation of flavonoids within suitably tailored nanocarriers may be a solution for these limitations and for achieving better therapeutic efficacy in the management of brain tumors.

Carotenoids are a class of naturally occurring dietary antioxidants with significant chemopreventive and chemotherapeutic potential in different types of cancers including CNS tumors. Carotenoids exhibit chemopreventive effects by suppressing the harmful effects of free radicals that regulate cancer cell proliferation, cell cycle progression, invasion, inflammation, and angiogenesis by regulating several molecular events including Akt/PI3K/mTOR, cyclin/cyclin-dependent kinase (CDK), PPAR, Wnt, VEGF, MMPs, and NF-κB signaling [207]. Like other antioxidants, carotenoids can also promote ROS production with prooxidant chemistry that aid in their chemotherapeutic potential. Lycopene is known to act on tumor cells by preventing DNA damage, suppressing survival, and inhibiting motility. In a preclinical assay, lycopene has shown the potential to induce apoptosis in glioblastoma cells [208]. Lycopene supplementation potentiates the therapeutic response to standard therapy in GBM by suppressing tumor recurrence [209]. In a randomized placebo control study, concomitant lycopene treatment in post-operative high-grade glioma patients receiving radiotherapy and chemotherapy with paclitaxel has shown the prospective therapeutic value as an adjuvant in the management of brain cancer [210]. Crocetin is a naturally occurring dicarboxylic acid apocarotenoid that exhibits a chemopreventive effect on glioblastoma cells by inhibiting proliferation and inducing morphology changes that is mainly mediated through activation of neuronal markers (class III β-tubulin and neurofilament) and suppression of mesenchymal markers (cluster of differentiation 44/90, octamer-binding transcription factor 3/4, and C-X-C chemokine receptor type 4) in glioma cells. In addition, crocetin imparts epigenetic modulation in glioma cells through the suppression of class I histone deacetylases (HDACs). Crocetin has also been shown to induce apoptosis by downregulating fatty acid synthase and cluster of differentiation (CD) 44 suppression and caspase 3 activation in glioblastoma cells and prevents cell migration. In in vivo tumor xenograft model, crocetin significantly inhibited glioblastoma tumor growth in female mice proposing its potential therapeutic attributes in brain tumor management [211]. Astaxanthin, a xanthophyll carotenoid exhibits potential anti-cancer effects by both chemopreventive and chemotherapeutic mechanisms. At 10 μM, it can suppress the growth and migration of glioblastoma cells by suppressing Erk1/2, Akt, cyclin D1, MMP2/9, and fibronection activation and activating p38 and p27 expressions. This chemopreventive effect is thought to be mediated through an antioxidant mechanism. In an in vivo study, astaxanthin also showed tumor inhibitory effects in terms of tumor area and volume in a murine glioblastoma model. Adonixanthin is an intermediate of astaxanthin that exhibits better chemopreventive quality on glioblastoma in both in vitro and in vivo studies compared with astaxanthin [212]. Astaxanthin also exhibits a chemotherapeutic effect on brain tumor via prooxidant effect by triggering intracellular ROS levels and subsequently endorses apoptosis in cancer cells. Interestingly, the prooxidant effect of astaxanthin is highly concentration dependent. At high concentrations (20–40 μM), it promotes apoptosis in different types of human astroglioma cells; however, at low concentrations (4–8 μM), it causes hormesis by promoting the cell cycle progression through CDK activation and increasing proliferation through the suppression of p53 antitumor protein [213]. In a recent report, hormesis at low doses has been revealed to be associated with the antioxidant properties of astaxanthin, and partly by lowering the mitochondrial membrane potential [214].

Coenzyme Q10 (CoQ10) is a lipid-soluble quinone involved in radical scavenging, mitochondrial electron transport, and membrane stabilization. Along with direct radical scavenging effects, it also helps to recover antioxidant vitamins from their oxidized states to aid in overall antioxidant effects [215]. In addition, CoQ10 can regulate several genes that are involved in different cellular processes [216]. A growing body of evidence shows that CoQ10 prevents cancer growth and proliferation by rewiring cancer metabolism [217]. CoQ10, along with other antioxidants, has been reported to improve the survival of end-stage cancer patients [218]. It can potentially sensitize human glioblastoma cells towards ionizing radiation and TMZ-induced cytotoxicity without imparting any cytotoxic effects to noncancerous cells. It switches to a prooxidant state and potentiates radiation-induced O_2_^•−^ and H_2_O_2_ accumulation that primarily happens due to a decline in CAT and SOD2 levels. It also suppresses HIF-1α, accompanied by decreased levels of lactate and other important metabolites that are involved in GSH synthesis [219]. Emerging evidence shows that radiation therapy followed by CoQ10 treatment synergistically eliminates glioma cell proliferation by remodeling the glial fibrillary acidic protein network [220]. Additionally, CoQ10 along with TMZ synergistically prevent the proliferation of murine glioma cells. It exhibited promise to suppress the invasion of TMZ-resistant rat glioma in both in vitro and in vivo models by suppressing *MMP9* gene and EMT markers, such as N-cadherin and vimentin proteins. However, in contrast to the observation of Frontiñán-Rubio and colleagues [219], this study claimed that the anti-invasive effect of CoQ10 is associated with an antioxidant mechanism mediated through the upregulation of SOD2 [215]. A recent report revealed that glioma cells differentially respond to high concentrations of oxidized CoQ10 (ubidecarenone) as compared with non-cancer cells. Oxidized CoQ10 arrests the cell cycle (G2/M phase) and prevents the proliferation of glioma cells without affecting normal cells and this chemopreventive effect was found to be associated with the enhanced production of intramitochondrial O_2_^•−^ specifically in glioma cells [221]. Despite preclinical studies showing the therapeutic promise of CoQ10 against brain cancers, no reports of randomized clinical trials are available so far regarding clinical use of CoQ10 in brain cancer patients.

Plumbagin (5-hydroxy-2-methyl-1,4-napthoquinone) is a naturally occurring antioxidant that exhibits an array of therapeutic attributes including anticancer effects against brain malignancies [222]. Plumbagin has been shown to inhibit the growth, migration, and invasion of brain tumors as well as endorses apoptosis by regulating several signal transduction pathways [222]. Plumbagin acts as a suppressor of forkhead box M1 (FOXM1), an oncogenic factor in different brain tumor cells [223, 224]. It arrests the cell cycle (G2/M phase) by CDK2/4 downregulation and triggers ROS production, leading to activation of apoptotic cell signaling in glioblastoma cells [225–227]. In addition, plumbagin is known to prevent brain cancer progression and metastasis by downregulating PI3K/Akt/mTOR signaling and suppressing MMP2/9 activation [222, 225]. Plumbagin can induce DNA damage and apoptosis by interfering with the telomere dynamics in human GBM cells [227].

Garlic-derived organosulfides, such as diallyl sulfide, diallyl disulfide, and diallyl trisulfide represent a class of sulfur-containing antioxidants of natural origin. These compounds can suppress the activation of carcinogens, endorse phase 2 detoxification processes, arrest the cell cycle, induce the intrinsic apoptotic pathway, and promote histone acetylation [228]. These compounds have been found to trigger apoptosis in human glioblastoma cells by promoting ROS production, activation of MAPKs and cysteine proteases, inducing ER stress, increasing intracellular Ca^2+^, upregulating calreticulin, and lowing mitochondrial membrane potential. In comparison with diallyl sulfide and diallyl disulfide, diallyl trisulfide is effective in killing human glioblastoma cells at a considerably lower dose and is the most potent of these three organosulfides [229]. Diallyl trisulfide also exhibited potential antitumor effect in a murine glioblastoma model by targeting multiple transcription factors without causing systemic toxicity. In an in vivo model, it showed the potential to arrest the cell cycle, suppress mitosis in tumor, endorse apoptosis, down-regulate pro-survival transcription factors (survivin, Bcl-2, phospho-Akt, c-Myc, mTOR, EGFR, and VEGF), and increase p21/WAF1 activation [230]. According to a recent study, diallyl disulfide is capable of causing cytotoxicity in a variety of human astrocytoma cells [231]. S-allyl-L-cysteine, another antioxidant garlic constituent has been shown to induce apoptosis in neuroblastoma cells [232]. Garlic-derived antioxidants have exhibited promise against brain tumor cells but more experiments are required for further clinical translation.

In this section, we discussed various naturally occurring antioxidants that exhibited potential for brain cancer treatment. Some of the molecules sensitize cancer cells to normal cancer treatments and other impart synergistic effects with other chemotherapeutic drugs. However, their precise roles like chemoprevention and chemotherapy need to be adequately addressed with respect to understanding the delicate antioxidant-prooxidant balance along with other governing factors. The negotiation between proapoptotic and prooxidant mechanisms in cancer chemotherapy, and intercession between antioxidant mechanism and anti-tumorigenic effects are critical issues in this aspect. Antioxidant effects could compromise chemotherapeutic effects and prooxidant effect may potentiate tumorigenesis. Thus, before utilizing antioxidants as therapeutic agents against brain cancer, these points require serious attention.

## Protective roles of antioxidant-rich diet in brain tumors

Already we discussed the protective roles of different naturally occurring antioxidants in brain cancers. Many of them are present in different foods. In addition, foods also contain trace elements that exhibit direct or indirect antioxidant effects. Considering the role of OS in tumorigenesis, these foods may offer chemopreventive roles by slowing cancer progression. Emerging evidence reveals an inverse association between an antioxidant-enriched diet and cancer risks [233, 234]. In this section, we will discuss the anti-tumorigenic effects of some antioxidant-enriched foods in brain tumor as reported in different case-control studies, cohort studies, prospective studies, and meta-analyses, as well as a few preclinical studies.

Dietary intake of antioxidant vitamins not only improves the survival of malignant glioma patients in advanced stages but also lowers the risk of newborms developing pediatric brain tumors when mothers routinely took these antioxidants during pregnancy [53, 54]. In a case-control study in eastern Nebraska, glioma risk in adults was found to be negatively correlated with the consumption of dark yellow vegetables, such as carrots, mixed vegetables containing carrots, yams, or sweet potatoes [235]. In an international case-control study comprising 1548 cases and 2486 controls, yellow-orange and leafy green vegetables (but not cruciferous) were shown to be negatively related to glioma risk [236]. Carotenoids present in dark yellow vegetables are thought to play a part in this chemopreventive function [235, 236]. In a case-control study in the San Francisco Bay Area, it has been shown that a higher intake of foods enriched with antioxidants and certain phytoestrogens, especially daidzein protects against the development of gliomas. This observation clearly suggests that these foods attenuate OS that plays a key role in gliomagenesis [237]. The daily antioxidant intake from food items is calculated by referring to the antioxidant index database for different food items [238]. In a meta-analysis comprising 15 studies with 5562 cases, Li attempted to understand the relationship between vegetable intake and glioma risk [71]. Seven of these studies reported a negative correlation between vegetable intake and glioma risk, whereas the remaining studies found no evidence of this relationship. After executing a comprehensive review of all studies, Li proposed an inverse relationship between vegetable consumption and the risk of glioma. However, the meta-analysis of 17 studies comprising 3994 cases suggested that fruits may have a protective impact on glioma among Asians but not in others. The presence of antioxidants present in vegetables and fruits may have a significant role in the claimed chemopreventive effect [71]. A meta-analysis of 12 studies comprising 1,960,731 participants along with 2987 glioma cases showed that every single cup of tea or coffee per day reduces the risk of glioma to 3%. Presence of antioxidants especially polyphenols in tea or coffee may contribute to the overall chemopreventive effect against glioma [239]. Selenium is an important micronutrient with antioxidant capacity. Preclinical studies showed that dietary supplements of selenium-enriched yeast could mitigate brain tumor growth and metastasis, thus prolonging the survival rate of brain tumor-bearing mice [240, 241]. In contrast, DeLorenze and colleagues reported that the association between daily dietary antioxidant consumption and survival in people with malignant gliomas is highly erratic and may differ depending on the histological group [92]. A similar observation has been cited earlier, where Salganik and peers experimentally revealed that dietary antioxidants had no impact on brain tumor growth, or could worsen the outcome [242]. Analysis of 3 large cohort studies in the US also failed to reveal any association between the average intake of fruits, vegetables and carotenoids and the risk of glioma in both men and women [243]. Zn is an essential micronutrient with potential antioxidant property, but Zn-enriched food did not exhibit any correlation with glioma risks [233, 244]. Therefore, serious attention is required to establish the correlation between the quality and quantity of foods and risk of brain cancer in different stages. More preclinical mechanistic studies are required to solve this puzzle.

## Effect of antioxidants on metabolic reprogramming

**Fig. 6:**
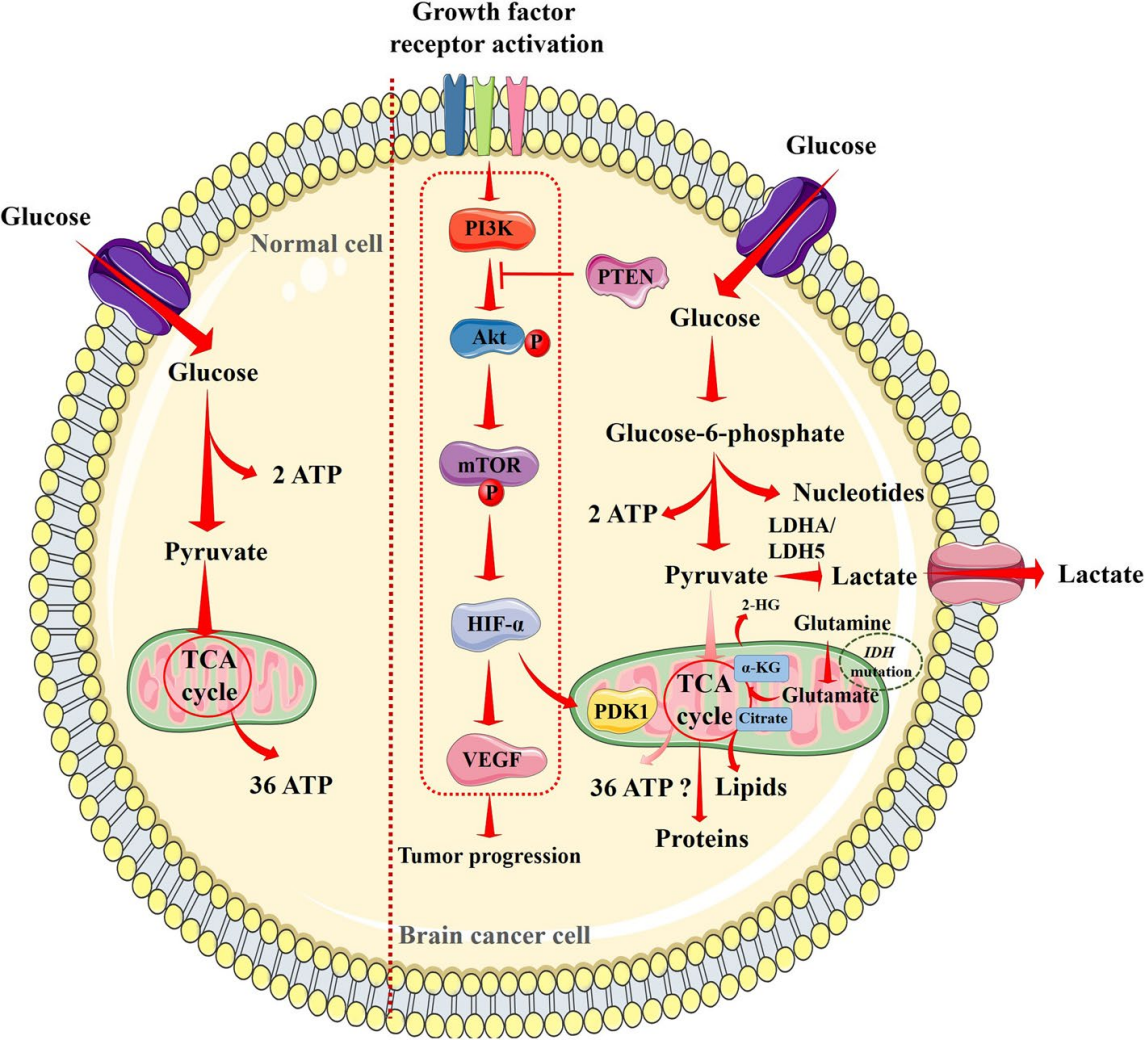
Metabolic reprogramming in brain cancer cell as compared with the normal cell. Brain tumor cell depends on glycolysis for glucose oxidation instead of oxidative phosphorylation and uses more glutamine. Cancer cells predominantly convert glucose into lactate even in the abundance of oxygen; this is referred to as aerobic glycolysis or the Warburg effect. The lactate aids in acidifying the tumor microenvironment and endorses invasion. Glioma cells also utilize glutamine as a substrate to enter the TCA involving IDH. Mutation of the IDH gene potentiates the formation of oncogenic 2-HG from α-KG. The metabolic reprogramming is executed by activation suppression of several transcription factors. Red arrows indicate downstream events and red lines indicate inhibition. 2-HG, 2-hydroxyglutarate; Akt, protein kinase B; ATP, adenosine triphosphate; HIF-1α, hypoxia-inducible factor 1-alpha; HK2, hexokinase 2; HK2, hexokinase 2; IDH, isocitrate dehydrogenase; LDH5, lactate dehydrogenase 5; LDHA, lactate dehydrogenase A; mTOR, mammalian target of rapamycin; PDK1, pyruvate dehydrogenase kinase 1; PI3K, phosphatidylinositide 3-kinases; PTEN, phosphatase and tensin homolog; TCA, tricarboxylic acid; VEGF, vascular endothelial growth factor; α-KG, α-ketoglutarate

Numerous metabolic changes have been found in brain cancer cells or cancer-initiating cells that ensure proper energy supply to endorse proliferation and invasion (Fig. 6). To cope with hypoxia, nutrient deficiency, or the nature of tumor microenvironment, the metabolic scheme of glioma cells is changed. Certain metabolic changes are involved in cancer initiation, while others contribute to cancer progression [245]. The two most typical changes, such as depending on glycolysis for glucose oxidation instead of oxidative phosphorylation and increased use of glutamine are seen in most cancers including brain cancer [246, 247]. The absence of oxidative phosphorylation reduces energy (ATP) production and enhances the accumulation of lactate that may aid in acidifying the tumor microenvironment and endorsing invasion [247]. Metabolism of glucose into lactate is the signature of glioma metabolic remodeling [248]. Glioma cells utilize glutamine as a substrate to enter the tricarboxylic acid cycle involving isocitrate dehydrogenase (IDH). Mutation of *IDH1* and *IDH2* genes may be associated with metabolic reprogramming by endorsing the conversion of α-ketoglutarate to 2-hydroxyglutarate, an oncometabolite. *IDH1* mutation causes reprogramming of pyruvate metabolism resulting in suppression of glucose oxidation via pyruvate dehydrogenase phosphorylation [249]. Glioma cells use fatty acids as major bio-energetic substrates. Fatty acid metabolism yields acetate that contributes half of the oxidative activities in glioma cells, while glucose contributes only one-third. In cancer cells, glucose is converted into fatty acids by the enzymatic action of fatty acid synthase. Activation of fatty acid synthesis and fatty acid oxidation is a signature of glioma cells [248]. The metabolic reprogramming is executed by activation of several transcription factors, such as HIF, HK2, lactate dehydrogenase 5 (LDH5), lactate dehydrogenase A (LDHA), PDK1, PI3K, Akt, mTOR, EGFR, etc., which induce the Warburg effect, suppress oxidative metabolism in mitochondria, limit pyruvate entry into the tricarboxylic acid (TCA) cycle, trigger anabolism, and endorse lactate formation. The suppression of PTEN, IDH, acetyl-CoA carboxylase, and different glycolytic enzymes are also implicated in metabolic reprogramming in brain cancer [250, 251]. Several naturally occurring antioxidants are implicated in reciprocation of metabolic reprogramming and limit cancer cell growth and survival by regulating the aforementioned transcription proteins. We have discussed the regulatory roles of different dietary antioxidants in section 5. In this section, we will discuss some other nature-derived antioxidant molecules that could have the potential to inhibit the growth and invasion of brain cancer cells by reinstating metabolic remodeling. Melatonin, a naturally occurring antioxidant simultaneously presents in animals as a pineal gland hormone that exhibits chemopreventive role against brain cancers [252]. Emerging evidence reveals that most of the melatonin enters into cancer cells through the glucose transporter, remodels glucose metabolism, and suppresses nutrients uptake by cancer cells [253]. In addition, it is thought to increase mitochondrial oxidative phosphorylation and reduces electron leakage through its antioxidant action. Thus, the chemopreventive effect of melatonin against brain cancer could be associate with its interference with metabolic reprogramming in brain cancer cells. Gossypol, a polyphenol of *Gossypium hirsutum* inhibits LDH5 that is involved in the conversion of pyruvate to lactate under the anaerobic milieu [251, 254]. Myricetin, a flavonoid has been shown to endorse glycolytic metabolism by activating sirtuin 3 (SIRT3). SIRT3 activation consequently suppresses PI3K/Akt signaling probably by activating PTEN resulting in the induction of apoptosis of glioblastoma cells [251, 255]. Petunidin-3-O-glucoside, an antioxidant anthocyanin glycoside of red grapes is also involved in the reciprocation of metabolic reprogramming in glioblastoma cells by the same mechanism [256]. Oxymatrine, a naturally occurring antioxidant alkaloid is also known to exhibit a chemopreventive effect against human glioblastoma cells by inducing apoptosis, suppressing invasion, and arresting the cell cycle by suppressing PI3K/Akt/mTOR signaling and downregulating STAT3 and EGFR activation, thereby counteracting with metabolic remodeling in brain cancer cells [251, 257]. Oleuropein, the principal phenolic aglycone of secoiridoid glycosides suppresses glioma cell growth and invasion by suppressing PI3K/Akt signaling [251, 258]. Phloretin, an antioxidant flavonoid abundant in many plant species is known to arrest the cell cycle and induce apoptosis by counteracting metabolic reprogramming in human glioblastoma cells mediated through activation of p27, downregulation of PDKs and cyclins, and suppression of PI3K/AKT/mTOR signaling. However, the effect is predominantly executed through a prooxidant mechanism provoked by enhanced ROS production. The antioxidant effect of N-acetyl-L-cysteine and glutathione is negatively associated with the reversal of metabolic remodeling by phloretin [251, 259]. Thus, the delicate prooxidant/antioxidant balance of an antioxidant executes a regulatory impact on cancer cell metabolism to impart chemopreventive role.

## Effect of antioxidants on gene mutation in brain cancer

Two major classes of genes, oncogenes and tumor suppressor genes, are important regulators of cancer biology. Oncogenes encode proteins that potentiate tumorigenesis by stimulating proliferation and regulating biological activities crucial for invasion, angiogenesis, migration, and other features of malignancy. In contrast, tumor suppressor genes encode proteins that are involved in antiproliferative, anti-invasive, anti-angiogenic, cell cycle arrest and apoptosis-inducing effects. Mutation of tumor suppressor genes or predisposition of modifier gene alleles has been regarded as a biological risk factor of tumorigenesis [260]. Tumorigenesis involves the accumulation of multiple genetic mutations in cells. ROS contributes to DNA methylation and damage, which results in mutations that turn healthy cells into cancerous cells [261]. Hypermethylation of tumor suppressor genes including MutL homolog 1 (*MLH1*), breast cancer 1 (*BRCA1*), FA complementation group F (*FANCF*), and checkpoint with forkhead and ring finger domains (*CHER*) is implicated in cancer development. Different antioxidants have been reported to counteract DNA hypermethylation by multiple mechanisms, including epigenetic regulation and chromatin remodeling process [262]. Vitamin C is known to inhibit TETs, and thereby endorses DNA demethylation to inhibit carcinogenesis through an epigenetic regulatory mechanism [263]. Vitamin E can also decrease DNA damage epigenetically by restoring the expression of DNA repairing genes including *DNA methyltransferase 1* (*DNMT1*) and *MLH1* [264]. Mutation of tumor suppressor gene, *p53* (*Tp53*)-residing short arm of chromosome 17 is implicated in astrocytoma and glioma. The *p53* gene encodes the p53 protein which acts as a negotiator of several events, including apoptosis induction, cell cycle arrest, and DNA repair after damage [260, 265]. Mutation of *p53* gene is implicated in an enhanced mortality rate of brain tumor patients [54]. Dietary antioxidants including vitamin E and C, flavonoids, curcumin, caffeine, resveratrol, etc. play a key role in cancer chemoprevention by restoring p53 activity [266]. It is hypothesized that, p53 activity aids in maintaining genome stability and acts as a “genome guard” against mutations that support carcinogenesis. However, the precise regulatory mechanism is yet to be specifically revealed [266]. *PTEN* gene, a tumor suppressor located at chromosome 10 encoding the PTEN protein, acts as a negative regulator of Akt. Mutation of *PTEN* is evident in glioblastoma cells [267]. Vitamin E, carotenoids, curcumin, resveratrol, genistein, xanthohumol, and garlic-derived organosulfides can suppress Akt activation via phosphorylation [107, 133, 158, 205, 207, 222, 223]. Different antioxidants, such as epigallocatechin gallate, genistein, carotenoids, resveratrol, stilbenes etc. were shown to upregulate PTEN expression which may be associated with the regulation of *DNMT1* transcription [262]. Thus, antioxidants may exhibit a chemopreventive role by restoring PTEN activities. Mutation of *CDK inhibitor 2A* (*CDKN2*) located on chromosome 9 is frequently seen in astrocytic neoplasms. *CDKN2* encodes p16 that inhibits CDK4, resulting in activation of retinoblastoma 1 (*RB1*), another tumor suppressor gene. *RB1* is involved in the suppression of the cell cycle process [268]. Antioxidants like resveratrol, curcumin, flavonoids, retinoids, etc. can induce p16 activation in different types of cancer cells [269–274]. Preclinical data strongly suggest that they could reciprocate methylation or mutation-mediated inactivation of p16 and could be beneficial as chemopreventive agents in cancer therapy. Mutation of *IDH* gene encoding IDH enzymes (IDH1/2/3) are frequently found in different types of cancer including brain cancer [275]. OS induced by carcinogens is thought to contribute to IDH1 inactivation. SOD2 can reciprocate IDH1 suppression, thus exhibiting a potential therapeutic approach [276]. Mutation of the *GST* gene encoding an antioxidant enzyme GST has been revealed to be associated with increased glioma risk. *GST* variants are linked to increased glioma risk differentially in different ethnic groups. The *GSTP1* Ile105Val variant increases overall glioma risk; *GSTP1* Ala114Val and *GSTT1* null/present variants are shown to increase the risk of glioma in Caucasian people, but not in the Asian population [277]. Thus, pharmacological or genetic stimulation of GST may play a chemopreventive effect in brain tumor. Single nucleotide polymorphisms of OS-responsive genes, such as *CAT*, *SOD1*, *SOD2*, *SOD3*, *GPx1*, *NOS3*, and *PON1* have been found in adult brain tumor studies. Among them, the *Ala* variant of *SOD2* rs4880 and the C variant of *SOD3* rs699473 are found to be associated with brain tumor risk [278]. Thus, the pharmacological or genetic activation of SOD2 and SOD3 could mitigate the risk of brain tumors. The mutation of tumor suppressor genes is a key issue in oncogenesis of different types of cancers. Enhanced OS mediated by pro-oncogenic factors has been regarded as the key contributor to these gene mutations. Thus, antioxidants surely can hinder oncogenesis by restoring redox balance, preventing DNA damage, regulating DNA methylation status, and repairing damaged DNA. Additionally, antioxidants have been shown to suppress cancer progression by endorsing down-stream signaling events of the tumor suppressor genes.

## Discussions and future perspectives

OS is regarded as one of the key contributors to disturb brain homeostasis and is involved in carcinogenesis of different types of brain cancers [49]. Thus, antioxidants may act as tumor-growth suppressors by preventing OS caused by different oncogenic factors [135, 184, 196, 197, 207, 212]. Antioxidants negatively influence cancer initiation by endorsing DNA repair. Increasing antioxidant intake has been shown to deplete OS, accordingly, creating an energy crisis for preneoplastic cells, resulting in suppression of cell growth and activation of cell death pathways to impart chemopreventive effect during cancer progression pathway [279]. On the other hand, all exogenous antioxidants support prooxidation chemistry that can also trigger OS by promoting the release of ROS under certain conditions, which is essential to kill neoplastic cells by inducing different cell death pathways to exert chemotherapeutic effects [60, 146, 197–200, 208, 213, 259]. Induction of a high level of OS to cancer cells by triggering ROS production and/or suppressing endogenous antioxidants is thought to be a potential strategy in cancer chemotherapy. In this aspect, it is preferred to have a prooxidant effect as opposed to an antioxidant effect to demonstrate chemotherapeutic effects or at least, enhance the chemotherapeutic potential of common cancer treatments [280]. Thus, an exogenous antioxidant at a prooxidant dose could promote carcinogenesis by inducing OS in pre-neoplastic cells [281]. The roles of antioxidants in cancer reside on a delicate line of antioxidant or prooxidant mechanisms depending upon the objective of the treatment. Cancer stages, severity, and treatments could be the main determining factors in selecting the requirement of pro- or antioxidant effect of an exogenous antioxidant; tumor types and location may also be factors.

The inclusion of dietary antioxidants following standard cancer treatments often prolongs the life span of glioma patients. That may be attributed to the retardation of tumor recurrence through the chemopreventive role of antioxidants mediated through an antioxidant mechanism. The prophylactic roles of antioxidants against nonspecific organ toxicity caused by attenuating OS imparted by chemotherapeutic drugs can also be accountable for improving the life span of brain cancer patients [94]. Some antioxidants were found to sensitize cancer cells to normal cancer treatments and impart synergistic effects with standard radio- and chemotherapy [58–60, 82, 84, 89, 171–173]. Antioxidants at pharmacological doses are thought to impart prooxidant effects that synergize the chemotherapeutic potential of common cancer treatments. Moreover, few antioxidants have shown promise in reversing drug resistance to clinically useful chemotherapeutic drugs [94, 282]. All of these could be related to the chemosensitizing role of antioxidants via prooxidant chemistry. In contrast, sometimes dietary antioxidants worsen therapeutic outcomes in brain cancer patients when given along with chemotherapeutic drugs [279], which could be linked to the counteraction of the prooxidant effect of the chemotherapeutics. No effect of antioxidants on brain cancer outcomes seems to be associated with the pharmacokinetic and bioavailability issues of naturally occurring antioxidants, but it may impact their ability to reach the tumor site in the brain.

However, the differences in study results regarding the effects of different exogenous antioxidants either in dietary (low) or pharmacological (high) doses on brain cancer deter from understanding the precise role of antioxidants in cancer patients. Since an exogenous antioxidant exhibits anticancer effect by both antioxidant (chemopreventive) and prooxidant (chemotherapeutic) mechanisms, the dose is a critical factor. In general, at high/pharmacological doses exogenous antioxidants exhibit prooxidant effects; while, at dietary doses, they exhibit antioxidant effects. During concurrent treatment with chemotherapeutic drugs and radiation therapy, a prooxidant dose may be useful for achieving synergy. Dietary doses may antagonize normal cancer treatments by counteracting with OS which is required to kill cancer cells during radio- and chemotherapy. However, once standard cancer therapies have been completed, maintenance of brain cancer patients with routine dietary antioxidant supplements may be helpful to prolong their life span by minimizing chemotherapy-induced nonspecific organ toxicity and mitigating cancer recurrence via antioxidant mechanism. Thus, the association between prooxidant mechanisms and chemotherapy, and negotiation between antioxidant mechanism and chemoprevention are critical issues in this aspect. It is very important to understand the regulatory factors influencing antioxidant and prooxidant effects on brain cancer cells. The pharmacokinetic parameters and bioavailability of exogenous antioxidants are critical factors. Of note, the absorption of antioxidants from dietary sources often differs from the supplement of antioxidants as drugs/chemicals [147, 283]. Also, crossing the BBB to reach the tumor site is also a critical issue for antioxidants. Thus, these biopharmaceutical aspects clearly contribute to the therapeutic efficacy of antioxidants in brain cancer treatment. Finally, the types, locations, and stages of brain tumors, as well as the therapeutic regimen are also important factors to be considered to hypothesize the desired effect (pro/antioxidant) from an exogenous antioxidant in brain cancer treatment. These issues need to be addressed through extensive preclinical studies. Otherwise, we can never achieve consistent clinical outcomes with exogenous antioxidants as monotherapy or as adjuvants with standard cancer therapy in the management of brain cancer.

## Conclusion

Naturally occurring antioxidants exhibit both chemopreventive and chemotherapeutic roles by two completely opposite mechanisms. Through prooxidation chemistry, they endorse cell death pathways, thus can be useful as therapeutic agents or as adjuvants with standard cancer therapy. In contrast, dietary antioxidants could hinder tumorigensis and prevent tumor recurrence after standard cancer therapy via an antioxidant mechanism. Antioxidant effects also aid in improving the life span of brain cancer patients by suppressing chemotherapy-induced nonspecific organ toxicity. Thus, the intercession between prooxidant mechanisms in chemotherapy, and the relationship between antioxidant mechanism and chemoprevention are critical issues in this aspect. It is also worthy to mention that antioxidant effects could compromise chemotherapeutic effects, while prooxidant effects might potentiate tumor recurrence. Thus, the key line between pro- and antioxidant effects is the most important decider for the use of an exogenous antioxidant in terms of achieving the therapeutic effect against brain cancer as per the objective of treatment. The dose is thought to be a key determinant of prooxidant and antioxidant effects. Exogenous antioxidants at pharmacological doses could be useful for chemotherapeutic purposes, while at dietary doses, they might be useful in chemoprevention. The pharmacokinetic and biopharmaceutical features of exogenous antioxidants and their ability to cross BBB to reach tumor sites should also be considered to achieve the desired therapeutic effect in brain cancer treatment. Thus, there are several unanswered questions that need to be studied with extensive preclinical mechanistic studies to explore the specific roles of antioxidants with respect to types, forms and stages of brain tumors in order to achieve their clinical utility in brain cancer management.
